# THE MAKING OF A FOOTPRINT IN PROTEIN FOOTPRINTING: A REVIEW IN HONOR OF MICHAEL L. GROSS

**DOI:** 10.1002/mas.21632

**Published:** 2020-05-12

**Authors:** Alan McKenzie‐Coe, Raquel Shortt, Lisa M. Jones

**Affiliations:** ^1^ Department of Pharmaceutical Sciences University of Maryland Baltimore Baltimore Maryland 21201

**Keywords:** protein footprinting, higher order structure, hydrogen deuterium exchange, hydroxyl radical protein footprinting

## Abstract

Within the past decade protein footprinting in conjunction with mass spectrometry has become a powerful and versatile means to unravel the higher order structure of proteins. Footprinting‐based approaches has demonstrated the capacity to inform on interaction sites and dynamic regions that participate in conformational changes. These findings when set in a biological perspective inform on protein folding/unfolding, protein–protein interactions, and protein–ligand interactions. In this review, we will look at the contribution of Dr. Michael L. Gross to protein footprinting approaches such as hydrogen deuterium exchange mass spectrometry and hydroxyl radical protein footprinting. This review details the development of novel footprinting methods as well as their applications to study higher order protein structure. © 2020 The Authors. Mass Spectrometry Reviews published by John Wiley & Sons Ltd. Mass Spec Rev

## I INTRODUCTION

Mass spectrometry (MS)‐based proteomics has undergone radical changes since initial studies of proteins by electrospray ionization (ESI) and matrix‐assisted laser desorption ionization (MALDI). As new techniques were established, the field of proteomics expanded to analyze protein structure, conformations, and interactions (Kaur et al., 2019). Currently, the field of structural proteomics is experiencing tremendous growth, as newly developed methods, that compliment traditional methods, are continually being assimilated (Table 1). Despite the efforts that have been invested in proteomics and the promise of protein‐based therapeutics, it remains an inherently complex area of study. In particular, proteomics has the caveat that the macromolecules under investigation, namely proteins, can exist in various forms at different times depending on its environmental and physiological conditions (Chen, 2014). Consequently, much effort has been invested in developing methods that can characterize the higher order structure (HOS) of proteins (Do Kwon et al., 2015).

**Table 1:** A comparative chart of structural proteomics techniques

Comparison of structural proteomics techniques					
	HDX‐LC‐MS/MS	FPOP‐LC‐MS/MS	Cryo‐electron microscopy (EM)	X‐ray crystallography	NMR
Protein size	Any size	Any size	Large (>150 kDa)	Any size	Small (<40–50 kDa)
Sample state	Purified in buffer	Purified in buffer or native in cells	Frozen in its native state	Crystallized in lattice structure	Dissolved in water
Resolution	Residue‐Level	Residue‐Level	Near atomic	High (~2 Å)	High (>2.5 Å)
Advantages	Suitable for studying hydrogen bonding networks and protein folding dynamics, nm amounts of sample are required, capable of elucidating protein structure and conformational dynamics of individual proteins or large protein complexes, can identify protein binding or interaction sites, and applicable for examining intrinsic disorder	Irreversible nature of hydroxyl label, microsecond time scale of label, suitable for post‐label sample handling, applicable in‐cells, mM of sample required for in vitro studies, protein interrogation in native or near native states, suitable for studying protein folding, dynamics, protein interactions, and bindings sites, accessible experimental set up	Small amounts of sample are required, lenient on sample purity, and fast sample preparation	High atomic resolution can reveal the 3D position of atoms, well developed technique, applicable for model building molecular weight range is broad (if it can be crystalized) and suitable for macromolecular complexes	Proteins analyzed in solution, 3D structural elucidation of a protein in its natural state, high‐resolution, no major sample preparation needed, and can reveal molecular dynamics and interactions
Limitations	Deuterium label is reversable due to back exchange, pH‐dependent protease, low experimental temperatures required, minutes to hours’ time frame insufficient for studying faster folding dynamics, incompatible with post‐labeling sample handling, and limited spatial resolution	Quantitating the number of hydroxyl radicals imposed on the system in real‐time	Particles in a myriad of orientations, potential for high noise and radiation damage, resolution relatively low, not suited for biomolecules with low molecular weight, costly EM equipment, and direct dependent on EM techniques	High quality crystals required; which is not always possible for larger proteins, membrane proteins, or intrinsically disordered proteins, crystallization process may damage particles, large sample quantities required, 3D structure is one static form of the molecule, diffraction can be problematic, and complete experimental workflow can take years to complete	Unable to resolve proteins with large molecular weight, large quantities of purified sample are required, NMR equipment is costly to maintain, sample prep can be difficult, computational simulations may present challenges and some experiments are time consuming

3D, three‐dimensional; HDX, hydrogen deuterium exchange; FPOP, fast photochemical oxidation of proteins; LC‐MS/MS, liquid chromatography–mass spectrometry/mass spectrometry; NMR, nuclear magnetic resonance.

Conservation of noncovalent interactions is a prerequisite in maintaining the HOS of proteins necessary for biological function. Traditional ESI requires low pH for protonation, elevated temperatures, high voltage, and organic solvents for efficient ionization, all of which perturb nonbonded interactions, rendering it unsuitable to interrogate the native protein structure (Mitra, 2019). To overcome these disruptions to the physiological state, native ESI was developed. Native ESI or native MS is performed under physiological temperature and pH, under these mild conditions interactions from the solution phase are preserved (Leney & Heck, 2017). Further advancement to native MS includes the implementation of nano‐ESI which produces smaller droplets that are able to desolvate at room temperature and reduces the voltage needed for ionization (Mitra, 2019).

With the advent of native MS and advancements in MS technology, experiments could be performed *in vacuo* expanding the role of the mass spectrometer from solely detection. Traditionally, proteomics focused on a “bottom‐up” perspective that consists of proteolytic digestion of the protein prior to MS analysis. Alternatively, in a “top‐down” approach, fragmentation is performed on the intact gas‐phase protein ions in the MS. Top‐down proteomics can report on a protein and its various proteoforms that may originate from genetic variation, alternative splicing, or posttranslational modifications (Smith & Kelleher, 2013; Catherman, Skinner, & Kelleher, 2014). Coupling native ESI with top‐down proteomics preserves noncovalent interactions, allowing findings to be placed in a biological context, and increases the dynamic range of top‐down fragmentation from 30 kDa to over 100 kDa (Stoakes, 2019).

Native‐MS has also been coupled to ion mobility spectrometry (IMS). IMS, with the ability to separate ions according to their rotationally averaged collisional cross‐section (CCS), has developed into a useful tool in structural biology. IMS requires much smaller sample amounts than traditional biophysical techniques and lower purity requirements as the target ion can be selected online. While the principle of separation in an IMS experiments occurs as a function of CCS, some platforms can directly report an experimental collisional cross‐section. The experimental CCS can then be compared with a theoretical CCS determined using computational molecular dynamics (Lanucara et al., 2014).

Alternatively, the need to conserve the native state *in vacuo* is circumvented by performing modifications in‐solution and using the MS to measure these changes, since the modifications were conducted under native conditions observations reflect the native HOS of the protein. Within recent years, protein footprinting has emerged as a noteworthy in‐solution approach for investigating higher order protein structure. Protein footprinting has demonstrated the capacity to provide insights on interaction sites and dynamic regions that participate in conformational changes (Johnson, Di Stefano, & Jones, 2019). Residue‐level resolution can also be achieved by proteolytic digestion followed by liquid chromatography LC‐MS/MS analysis (i.e., *bottom‐up* approach). To date, protein footprinting has been employed to research the HOS of a plethora of large systems such as antibodies, large multi‐protein assemblies, viruses, membrane proteins embedded in micelles, nanodiscs, and intact cells (Baerga‐Ortiz et al., 2002; Lanman et al., 2004; Guan & Chance, 2005; Coales et al., 2009; Houde et al., 2009; Espino, Mali, & Jones, 2015; Lu et al., 2016; Watkinson et al., 2017; Zhu et al., 2017).

In this review, we will look at the contributions of Dr. Michael Gross to structural biology, specifically, in the field of protein footprinting. The Gross research group focuses on developing MS‐based methods to better understand the biophysics of proteins as it relates to their interfaces, interactions, folding and unfolding. This involves the development of novel technologies and methods to explore the interface and affinity between proteins and ligands, conformational changes in proteins in response to perturbation, and investigating the folding of proteins.

## II Interrogating HOS via MS‐Based Footprinting

**FIGURE 1:**
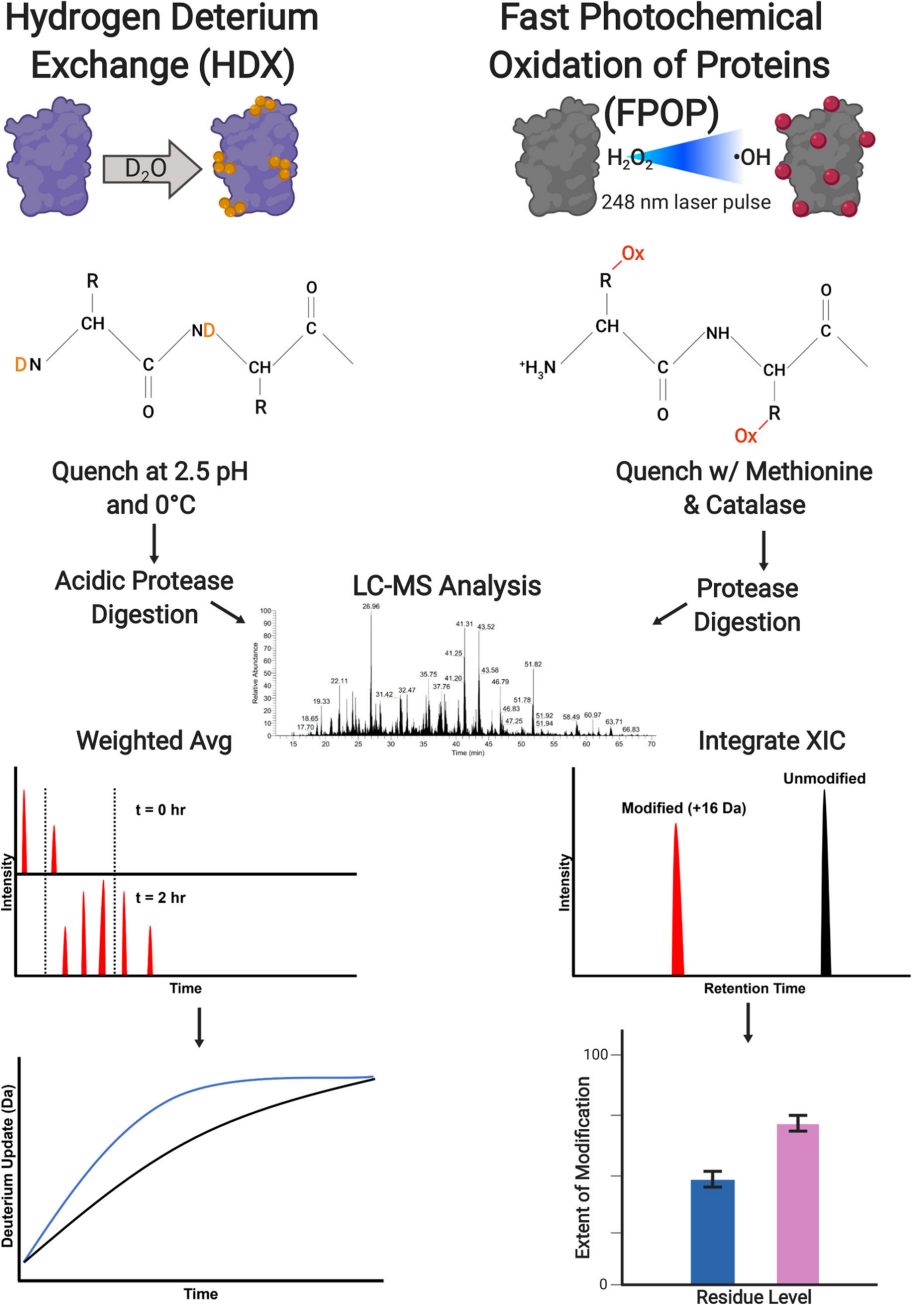
A schematic of working principles for HDX versus FPOP. Created with BioRender.com. LS‐MS, liquid chromatography–mass spectrometry. [Color figure can be viewed at wileyonlinelibrary.com]

The underlying principle behind MS‐based footprinting is that a chemical probe reacts with solvent accessible areas of the biomolecule producing a mass shift that is detectable by MS. Differential experiments are conducted under relevant structural states such as in the presence and absence of a ligand, or under native and denaturing conditions. Comparative analysis of the resulting labeling show which regions become buried, exposed or remain the same. Ultimately, this provides insight into protein folding/unfolding, protein–protein interactions, protein–ligand interactions, and conformational changes. Below we will discuss two footprinting methodologies in MS‐based structural proteomics that have prominently featured in Dr. Michael Gross’ research group, namely, hydrogen deuterium exchange mass spectrometry (HDX‐MS) and fast photochemical oxidation of proteins (FPOP) (Fig. 1).

### A HDX

Among the protein footprinting methods, HDX is unique in that it is not solely dependent on solvent accessibility differences but also on changes in hydrogen bonding, specifically, those involved in protein secondary structure. Originally measured by nuclear magnetic resonance (NMR) (Englander & Kallenbach, 1983), early work by Zhang et al. extended it for MS‐based studies, making it the first tool in the MS‐based footprinting toolbox (Zhang & Smith, 1993). Early work of the Gross group implementing HDX‐MS demonstrated the potential of the technique to be used as a quality control for recombinant protein drugs. This was shown in the case of four forms of insulin used for treatment of Type I diabetes, two of which are produced naturally from bovine and porcine, and two of which are synthetic. Previous studies demonstrated that variants of insulin can have different absorption and activity times (Koivisto & Felig, 1980; Fernqvist et al., 1986; Ramanathan et al., 1997). The HDX kinetics revealed differences in the tertiary structure of these four variants which contrasts with NMR studies where chemical shifts were nearly identical for the variants. This work asserted the power of HDX‐MS in detecting subtle tertiary structure differences which may be undetected by other methods.

**FIGURE 2:**
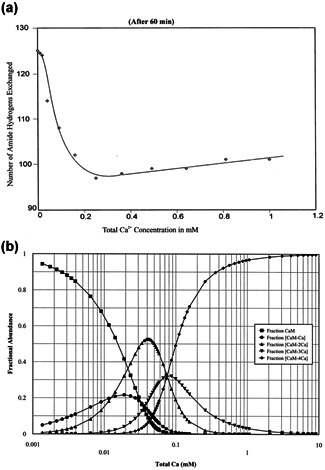
(**a**) Ca^2+^ titration curve for the H/D exchange of calmodulin. Note the break at approximately 0.25 mM added Ca21. The concentration of calmodulin was 15 µM. The solid line is the best fit as judged by eye. (**b**) Fractional species calculation for calmodulin‐*x*Ca^2+^ as a function of [Ca^2+^]. The total calmodulin concentration is 15 µM (Nemirovskiy, Giblin, & Gross, 1999).

Next, the focus was shifted to testing whether the method is useful in studying protein–ligand interactions using calmodulin as a model system. By investigating the conformational change of calmodulin upon calcium binding, the Gross lab showcased the utility of HDX‐MS to inform on protein–ligand interactions (Nemirovskiy, Giblin, & Gross, 1999). To do this, a titration experiment was performed by mixing fifteen micromolar calmodulin with increasing concentrations of calcium acetate followed by 60 min of deuterium exchange and ESI‐MS. The resulting titration curve showed three regimes (1) in low amounts of calcium (0.01 and 0.02 mM), there was no observable change in number of deuterium exchanged in comparison to the apo form; (2) at intermediate calcium concentrations (up to 0.25 mM), the anticipated increase in the amount of calcium‐bound calmodulin species resulted in a decrease in the number of deuterium exchanged, and (3) high concentrations of Ca^2+^ produced a slight upward trend in deuterium suggesting a reopening of the protein which was attributed to coulombic repulsion from nonspecific calcium binding (Fig. 2a). To help interpret the HDX data, known calmodulin‐Ca^2+^ binding constants were used to determine binding fractions for the different calcium‐bound species, calmodulin‐*x*Ca^2+^ (*x* = 0–4), with respect to total calcium concentration. The equations defining the binding fractions were then calculated and plotted using a calcium concentration of 10 nM to 0.10 M and a calmodulin concentration of 15 µM, the results of which could be used to accurately deduce the number of bound Ca^2+^ ions at a given total calcium concentration (Fig. 2b). The binding fraction data showed that at the calcium concentration corresponding to the least amount of deuterium uptake, or the most protected conformation, 85% of all calcium‐bound calmodulin are fully saturated. Quantitative characterization of the calmodulin‐2Ca^2+^ and the calmodulin‐4Ca^2+^ was of great interest as previous work reported cooperativity between the two globular domains of calmodulin during calcium binding (Greenlee, Andreasen, & Storm, 1982; Mills, Bailey, & Johnson, 1985; O'Neil & DeGrado, 1985; Iida & Potter, 1986). With insight from the binding fraction data, the calcium concentration was selected to generate the calmodulin‐2Ca^2+^ (0.04 mM) and the calmodulin‐4Ca^2+^ (0.49 mM) species. HDX kinetic curves, showing deuterium exchange as function of time, were constructed for apo‐calmodulin, calmodulin‐2Ca^2+^, and the calmodulin‐4Ca^2+^ species. The results of which show that the rate of exchange for the fully calcium‐bound calmodulin was less than that of apo‐calmodulin and the calmodulin‐2Ca^2+^ species. The lower deuterium exchange rate of the fully bound calmodulin was attributed to the protein adopting a more compact structure which lowers the number of solvent‐accessible hydrogens. This early study demonstrated the utility of HDX‐MS for studying protein–ligand interactions and the effect of ligand binding on the structure and dynamics of its target protein.

### B Protein‐Ligand Interactions in Solution by MS, Titration, and, H/D Exchange (PLIMSTEX)

Perhaps the most seminal work on HDX from the Gross group has been the development of PLIMSTEX. The PLIMSTEX workflow can be used to probe binding affinities, stoichiometries and conformational changes for a variety of protein–ligand interactions such as small molecules, metal ions, peptides, and proteins (Zhu et al., 2003a,b; Sperry et al., 2011). From a practical perspective, PLIMSTEX is compatible with various proteins, ligands, buffers, salt concentrations, pH, and temperatures.

**FIGURE 3:**
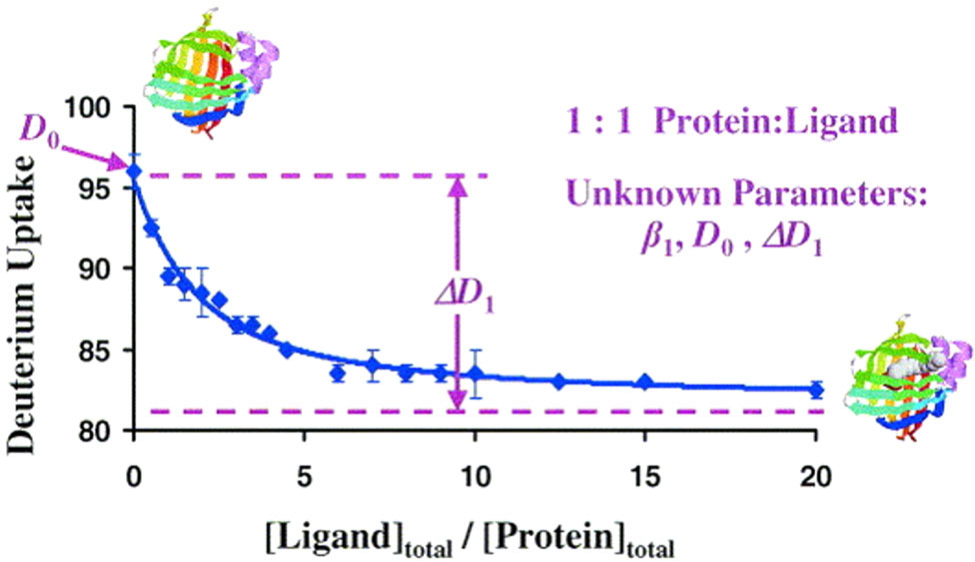
A typical PLIMSTEX curve for 1 to 1 protein:ligand binding. Adapted with permission from reference Zhu et al. (2004). PLIMSTEX, protein–ligand interactions in solution by MS, titration, and, H/D exchange. [Color figure can be viewed at wileyonlinelibrary.com]

In PLIMSTEX, the protein of interest is titrated with different concentrations of the ligand and subjected to H/D exchange after ligand equilibration, MS analysis is then used to determine deuterium uptake (Zhu, et al., 2003; Mei, Zhu, & Michael, 2007). A plot of the deuterium uptake versus the total ligand concentration results in a PLIMSTEX curve (Zhu et al., 2003a,b; Sperry et al., 2011). This curve is fitted with a 1:*n* protein–ligand sequential‐binding model, where *n* is the number of binding sites (Fig. 3) (Zhu, Rempel, & Gross, 2004). Information obtained from a PLIMSTEX curve is twofold. First, the binding constant of the protein interaction can be determined. Second, the deuterium shift can provide information on the structural effects of ligand binding. The deuterium shift, ∆*D*_*i*_ describes the difference between the average deuterium level of each ligand bound species and the apo‐form of the protein (Zhu et al., 2003a,b; Huang et al., 2011). A positive deuterium shift indicates that at *i* number of ligands bound the protein is better protected in comparison to the apo‐form. A negative deuterium shift is indicative of the protein becoming structurally extended relative to its apo form. Finally, a deuterium shift that is approximately zero indicates that there is an insignificant difference between the deuterium uptake of the protein–ligand complex and the apoprotein which suggests that there is no observable structural change.

In the study where PLIMSTEX was first introduced, Zhu et al. demonstrated the potential of the technique to be used to determine the binding constant, *K*, for four model protein–ligand interactions that have been previously reported (1) rat intestinal fatty acid binding protein (I‐FABP) and potassium oleate, (2) GDP‐bound human p21^H‐ras^ protein (Ras‐GDP) and Mg^2+^, (3) calcium‐saturated porcine calmodulin and melittin, (4) apo calmodulin and Ca^2+^ (Zhu et al., 2003a,b). The binding constants of these protein–ligand interactions were determined experimentally using PLIMSTEX and were within a factor of four of the literature values (Table 2) except for the holo‐CaM/melittin system. This discrepancy was attributed to a difference in experimental parameters. From this initial study PLIMSTEX showed the potential to be applied to determine the conformational change, binding stoichiometry, and affinity of protein–ligand interactions.

**Table 2:** Titration parameters obtained by PLIMSTEX. Adapted with permission from reference (Zhu et al., 2003a,b)

Protein (*C*_total_) + ligand (1 to *n*)	∆*D_i_*	PLIMSTEX *K_i_* (M^−1^)	Literature *K_i_* (M^−1^)
I‐FABP (0.3 µM) + oleate (1 to 1)	13.8 ± 0.7	(2.6 ± 0.2) × 10^6^	*K*_1_: 3.0 × 10^6^ (4.8 or 25) × 10^6^
Ras‐GDP (1.5 µM) + Mg^2+^ (1 to 1)	25.45 ± 0.07	(4.0 ± 0.3) × 10^4^	*K*_1_: 6.9 × 10^4^
Holo‐CaM (0.15 µM) + melittin (1 to 1)	29.3 ± 0.2	(5.44 ± 0.03) × 10^7^	*K*_1_: 33 × 10^7^
Apo‐CaM (15 µM) + Ca^2+^ (1 to 4)	14.1 ± 0.5	(5.4 ± 0.5) × 10^4^	*K*_3_: 3.98 × 10^4^
		(0.9 ± 0.1) × 10^5^	*K*_4_: 3.16 × 10^5^
		(5.0 ± 0.4) × 10^9^	*K*_3_ *K*_4_: 12.6 × 10^9^

PLIMSTEX, protein–ligand interactions in solution by MS, titration, and, H/D exchange.

**FIGURE 4:**
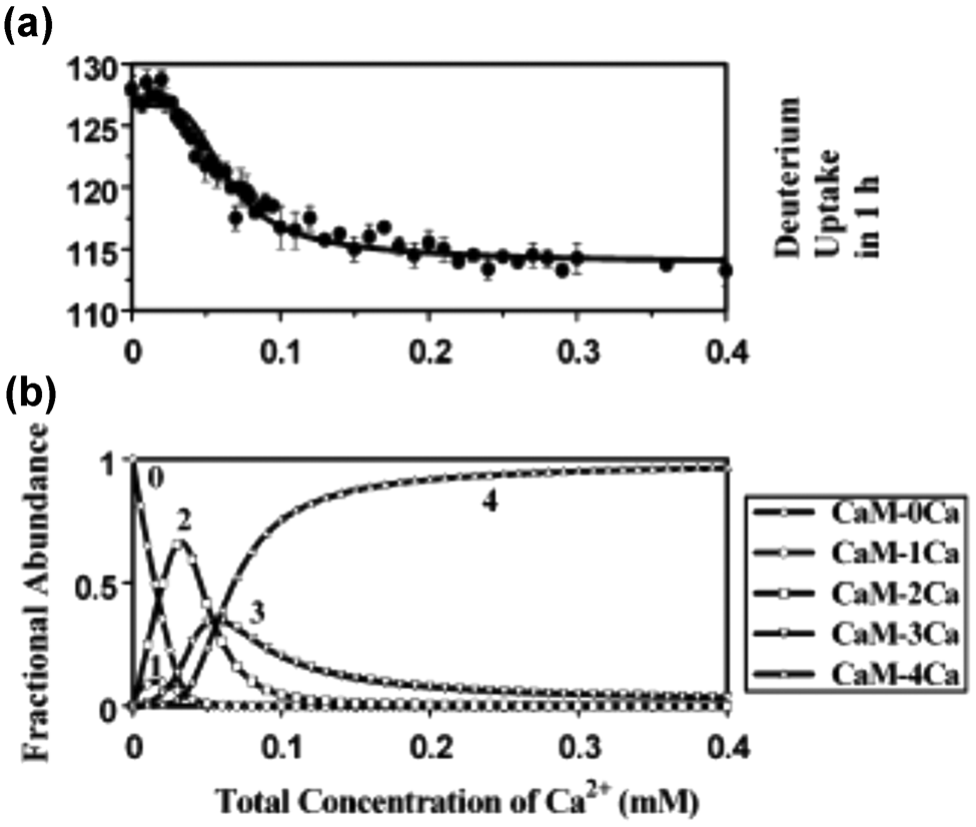
(**a**) Ca titration for 15 µM porcine calmodulin in 50 mM HEPES (pH 7.4, T 21.5°C, 90% D2O). Error bars are based on the deviation from two sets of Q‐TOF data. The solid curve is the best fit for the average data using the four‐ parameter model. (**b**) Species fraction as a function of total [Ca^2+^] for calmodulin interacting with four Ca^2+^. Adapted with permission from reference Zhu et al. (2003a,b). HEPES, 4‐(2‐hydroxyethyl)‐1‐piperazineethanesulfonic acid; Q‐TOF, quadrupole time‐of‐flight.

The first application of PLIMSTEX (Zhu et al., 2003a,b), which utilized an improved HDX protocol that incorporated the use of a guard column, revisited the titration of calmodulin with calcium. In this study, the acquired PLIMSTEX data was coupled to binding fraction calculations, as in earlier calmodulin‐based studies, to determine the number of bound Ca^2+^ ions. Comparison of the PLIMSTEX curve with the binding fraction calculations reveal that the titration curve behavior is governed by the calmodulin‐4Ca^2+^ species, given this insight, a 1:4 sequential‐binding model was used to fit the titration data (Fig. 4). Combining these results show that in transitioning from the apo to holo form, the first two calcium ions do not significantly affect the conformation and the calmodulin‐1Ca^2+^ and calmodulin‐3Ca^2+^ species quickly give way to the calmodulin‐2Ca^2+^ and calmodulin‐4Ca^2+^ forms. These observations substantiate that calcium binding by calmodulin is cooperative. Deuterium uptake as a function of time for calmodulin‐4Ca^2+^ was fitted with a three group pseudo‐first‐order kinetic model that parsed exchangeable amide hydrogens into “slow,” “intermediate,” and “fast.” The kinetic model made it possible to follow the distribution of type of hydrogen, i.e. fast, intermediate, or slow during HDX exchange kinetics, producing a fingerprint for the conformational changes of calmodulin during calcium binding. Experimentally determined binding constants from this study substantiate that calcium binding of calmodulin occurs cooperatively. This study also showed the robustness of the technique as it could determine the conformational changes, binding stoichiometry, and binding constants for Ca^2+^ interactions of calmodulin even in the presence of other metals and under various conditions of ionic strength.

Further refinements incorporated proteolytic digestion to achieve peptide level resolution of the structural and conformational changes that occur as a result of ligand binding. Subsequent work demonstrated the utility of PLIMSTEX to report on conformational change, binding stoichiometry, and affinity for various protein–ligand interactions where the ligand can be a small molecule (Wang et al., 2017), metal ions (Huang et al., 2011), and peptides (Sperry et al., 2011).

### C PLIMSTEX Variants

#### i Self‐association interactions using mass spectrometry, self‐titration and H/D exchange (SIMSTEX)

Subsequent work on PLIMSTEX focused on extending the technique to probe oligomerization of proteins. This approach, SIMSTEX is very similar to PLIMSTEX except that the protein is titrated with itself instead of a ligand (Chitta et al., 2006; Zhang et al., 2016). The first demonstration of SIMSTEX was performed on recombinant (r‐) human insulin, a 51 amino acid blood sugar regulating protein that consists of 2 chains (A and B) that are held together by disulfide bonds, and selected analogs and mutants. Insulin is stored in the pancreas as a hexamer but must dissociate to a monomer to be biologically active. Previous work proposed that longer activation times correlates to an increased propensity to self‐associate (Brange & Vølund, 1999). Structural studies via X‐ray crystallography shows that the B‐chain plays an important role in dimerization, specifically, nonpolar interactions involving residues at positions 23‐26, 28, and also by hydrogen bonding involving residues at positions 24 and 26 (Blundell et al., 1972; Baker et al., 1988). Additional structural investigations of hexamer insulin revealed that six glutamic acids residues located on chain B at position 13 are arranged in a circular pattern around a Zn‐binding atom that reduces charge repulsion (Brange & Vølund, 1999). Therapeutic strategies focus on generating mutants that more readily dissociate into the active monomer. Lispro, an Food and Drug Administration‐approved drug, differs from r‐human insulin by switching the proline at position 28 with the lysine at position 29 on the B‐chain. This single mutation causes a decrease in the dimerization constant. SIMSTEX was used to explore the structural implications of the ability of r‐human insulin, insulin analogs, lispro, and available mutants to oligomerize and dissociate (Table 3). SIMSTEX curves were generated by titrating the protein with itself and monitoring the change in deuterium uptake. On the basis of literature (Pocker & Biswas, 1981), SIMSTEX data was fit assuming association occurs sequentially by (1) monomer to dimer, (2) dimer to tetramer, and (3) tetramer to hexamer. However, the tetramerization constant of the SIMSTEX data was significantly low, so the data was also fit to a model that did not have a tetramerization step. This resulted in more accurate values for the affinity constants of dimerization and hexamerization. SIMSTEX data corroborates the fast‐acting nature of lispro as the observed dimerization constant was ~20 times smaller than r‐human insulin and it did not undergo hexamerization at the concentrations used. Analysis of the insulin mutants revealed that the mutants B9, B21, and B13B21 have significantly smaller oligomerization equilibrium constants than r‐human insulin. In the case of the insulin mutant B9, the reduced oligomerization was attributed to the addition of a charge site brought about by replacing a serine residue with an aspartic acid in chain B at position 9. The oligomerization of the B21 mutant, which has a glutamic acid residue on chain B at position 21 replaced with glutamine, is affected to the degree that no observable hexamer is formed. This difference was ascribed to the location of the mutation which occurs in a β‐turn that plays an essential role in many of the secondary structural features of the protein. The B13B21 mutant, which has the same mutation of B21, in addition to a mutation on the B chain of glutamic acid at position 13 to a glutamine residue regains the ability to undergo hexamerization. The reemergence of hexamer formation highlights the role of the glutamine residue in oligomerization and was credited to a decrease in charge repulsion at the center of the hexamer (Bentley et al., 1992). This work demonstrates the ability of SIMSTEX to report on self‐associating events of proteins in relevant biological conditions.

**Table 3:** Sequence variations for insulin analogs and insulin mutants (Chitta et al., 2006)

Insulin type	Sequence variation
Lispro	KB28 and PB29 reversed
Bovine	A in B30
Porcine	A in B30, A in A8, and V in A10
Insulin mutant	Mutation
A4A17	GluA4 and GluA17 to Gln and TyrB31
B27	ThrB27 to Arg
A4B27	GluA4 to Gln and ThrB27 to Arg
B9Asp	SerB9 to Asp
B21	GluB21 to Gln
B13B21	GluB13 and GluB21 to Gln

#### ii Dilution Strategy Protein‐Ligand Interactions in Solution by MS, Titration, and, H/D Exchange (dPLIMSTEX)

Dilution strategy PLIMSTEX is another variant of PLIMSTEX, and includes a dilution scheme to improve sample consumption (Tu et al., 2010). In particular, the method was developed to measure the ligand, in this case the peptide antigen, in an antibody‐antigen system. This stands in contrast to a normal PLIMSTEX experiment that follows the signal from the protein. The rationale behind the inverted approach is that the mass spectrometer can monitor the deuterium exchange of the ligand with higher accuracy and more precision than the large protein. In a typical dPLIMSTEX experiment, the protein‐peptide complex is allowed to equilibrate and the resulting sample is separated into two equal volumes (Tu et al., 2010; Zhang et al., 2016). One aliquot continues to HDX analysis, the other is diluted in aqueous buffer. The resulting diluted sample is divided in half with half of the volume again going to HDX analysis and the other half being further diluted. This procedure is repeated until the concentration of the peptide is no longer detectable.

**FIGURE 5:**
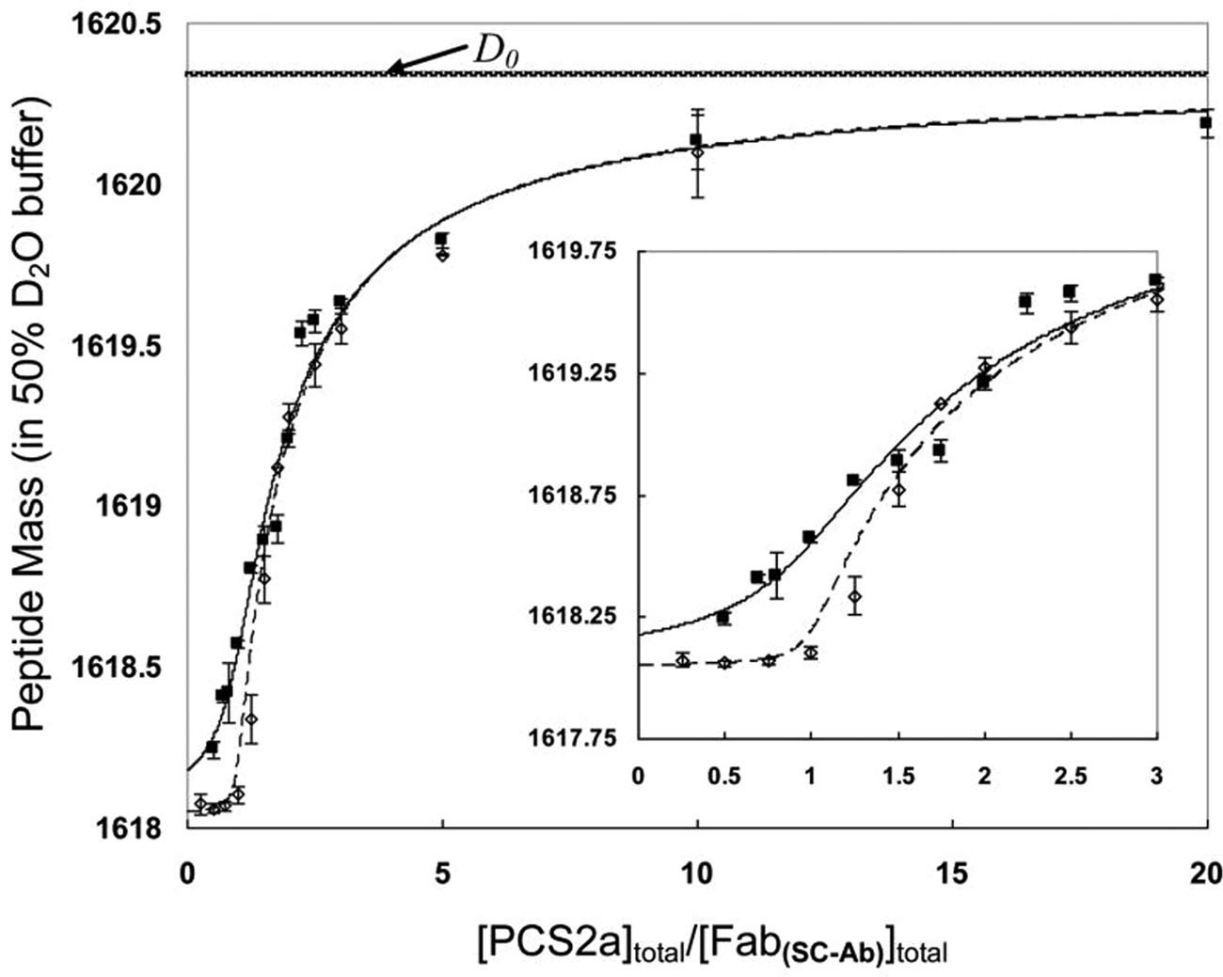
dPLIMSTEX fitting curves for SC‐Ab: PCS2a system. The concentration of antibody Fab regions (shown as [Fab _(SC‐Ab)_]) is used in place of the antibody concentration in the modeling. [Fab _(SC‐Ab)_]_total_ for the dashed line is 660 nM, whereas [Fab_(SC‐Ab)_]_total_ for the solid line is 41 nM. The error bars shown for the data points represent the standard deviation from duplicate independent experiments. The *D*_0_ mass is for the unbound peptide and is depicted as a line representing the asymptote for the other curves. Adapted with permission from reference Tu et al. (2010). dPLIMSTEX, dilution strategy protein–ligand interactions in solution by MS, titration, and, H/D exchange. [Color figure can be viewed at wileyonlinelibrary.com]

As a proof of concept, fully calcium‐bound calmodulin and the opioid β‐endorphin were used as a model system. The dissociation constant (4.9 ± 0.2 µM) for the calcium‐saturated calmodulin‐endorphin complex determined by dPLIMSTEX was in good agreement with the dissociation constant determined by traditional PLIMSTEX studies (4.5 ± 0.2 µM); both of which were comparable to reported literature values (Malencik & Anderson, 1982; Sellinger‐Barnette & Weiss, 1982). Once this method was validated using the model system, an antibody‐antigen system was analyzed. Affinity between an antibody and antigen can be as high as 10^8^–10^10^ M^−1^, taking advantage of this, the antibody‐antigen system, SC‐Ab: PCS2a, was used to demonstrate the sensitivity of the dPLIMSTEX platform. The results of which demonstrated that experiments could be performed at nanomolar concentration of antibody (41 nM) and peptide (20.5 nM) for the SC‐Ab:PCS2a system (Fig. 5).

PLIMSTEX and its variants make it possible to study a plethora of protein–ligand interactions under a variety of conditions (i.e. in buffer or in presence of metals). PLIMSTEX also shows versatility as it can also be tailored to study oligomerization processes in which the protein is titrated with itself. Ultimately, the PLIMSTEX platform provides a method to quantitively evaluate a protein's affinity for the titrant. Data obtained from these experiments can also be fit to kinetic models that provide a distribution on the rate of deuterium exchange.

### D pH‐Dependent HDX

Since HDX rates are highly dependent on pH, it has always been challenging to report on pH‐dependent structural changes. One such case is the diphtheria toxin, which enters a cell using the endosome pathway but must first undergo a conformational change induced by acidic conditions (Hoch et al., 1985). Conformational heterogeneity inhibited characterizing the structural transitions of the translocation domain during the pH‐induced refolding event. Forest and coworkers developed pH‐dependent HDX and demonstrated its utility to study the pH‐dependent structural changes of the translocation domain of the *Diphtheria* toxin (Man et al., 2010). By incorporating fungal protease type XVIII, which is active at acidic conditions, into the digestion workflow the digestion efficiency was increased resulting in increased proteome coverage (Cravello, Lascoux, & Forest, 2003; Man et al., 2010; Li et al., 2014).

**FIGURE 6:**
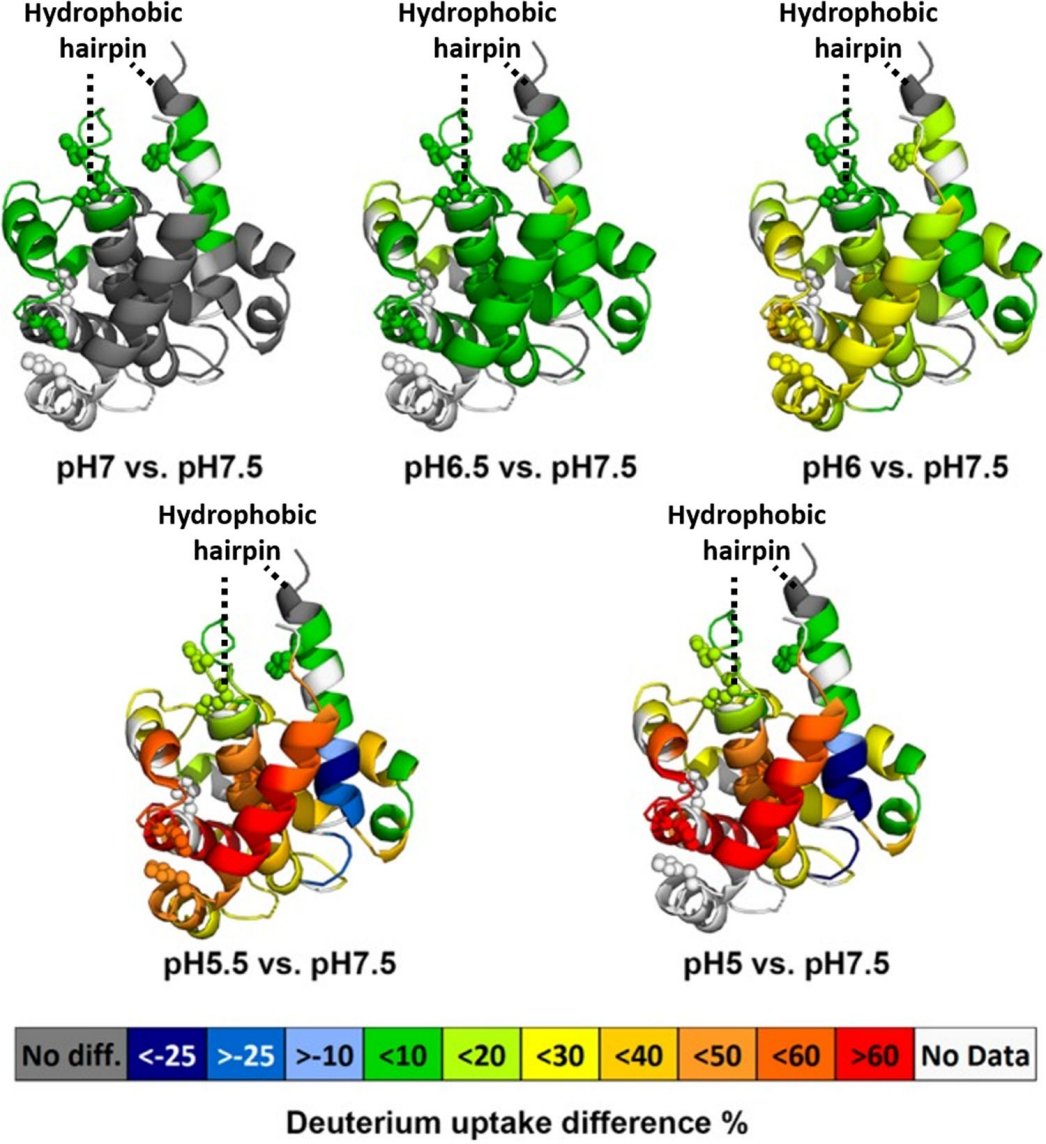
Averaged deuterium uptake differences between a low pH state and standard state (pH 7.5) mapped onto crystal structure of DTT (PDB: 1MDT). The color code (see legend) represents the differential HDX between a low pH condition and the standard pH condition; the regions in the crystal structure colored in white are not detected by HDX. Protonation of histidine residues (shown as spheres) is implicated in modulating pH‐dependent refolding of the T‐domain. Adapted with permission from reference Li et al. (2014).

In an extension of Forest's work on the diphtheria toxin, the Gross group conducted HDX experiments at several values of pH, from 5.0 to 7.5 at 0.5 intervals (Fig. 6), as opposed to just two pH values (5.0 and 7.5) (Li et al., 2014). To correct for the pH dependence of the deuterium exchange, the deuterium uptake was standardized to that of the pH 7.5 by modifying the exchange time accordingly. The results show that upon lowering pH, a transition from the native state to a membrane‐competent state occurs. The membrane‐competent state, unlike the native state, contains a solvent accessible hydrophobic hairpin which plays a role in membrane insertion. As the pH lowers, the protein fully unfolds resulting in full exposure of the hydrophobic hairpin allowing for translocation. Another observation of this study was that the T domain will oligomerize at low pH and in the absence of a membrane. This pH‐dependent aggregation was attributed to molecules of the protein interfacing at the exposed hydrophobic hairpins and also corroborated the proposed structural transition.

### E Pulsed HDX

Conformational heterogeneity also obstructs protein aggregation studies, as the protein will undergo conformational changes as a function of time. Despite the ability of HDX to overcome the challenges of structural heterogeneity, care must be taken in using HDX to study time‐dependent processes as HDX is also a time‐dependent process. Early work demonstrated the ability to detect folding intermediates by monitoring changes in the level of amide hydrogen exchange (Schmid & Baldwin, 1979; Kim & Baldwin, 1982). The approach would be developed into pulsed HDX by Englander and applied to protein folding intermediates (Roder, Elöve, & Englander, 1988; Pan, Wilson, & Konermann, 2005; Khanal et al., 2012). In traditional pulsed HDX, the protein is subjected to a denaturing D_2_O solution which results in the unfolded protein and deuterium exchange of accessible hydrogens. Refolding, performed by rapid dilution of the denaturing solution, is conducted at different time windows. As the protein refolds it occupies transient structural intermediates that are pulsed with H_2_O, residues of the protein that have not refolded remain solvent accessible and exchange back to hydrogen, whereas residues that have refolded are protected from the exchange event and retain deuterium.

**FIGURE 7:**
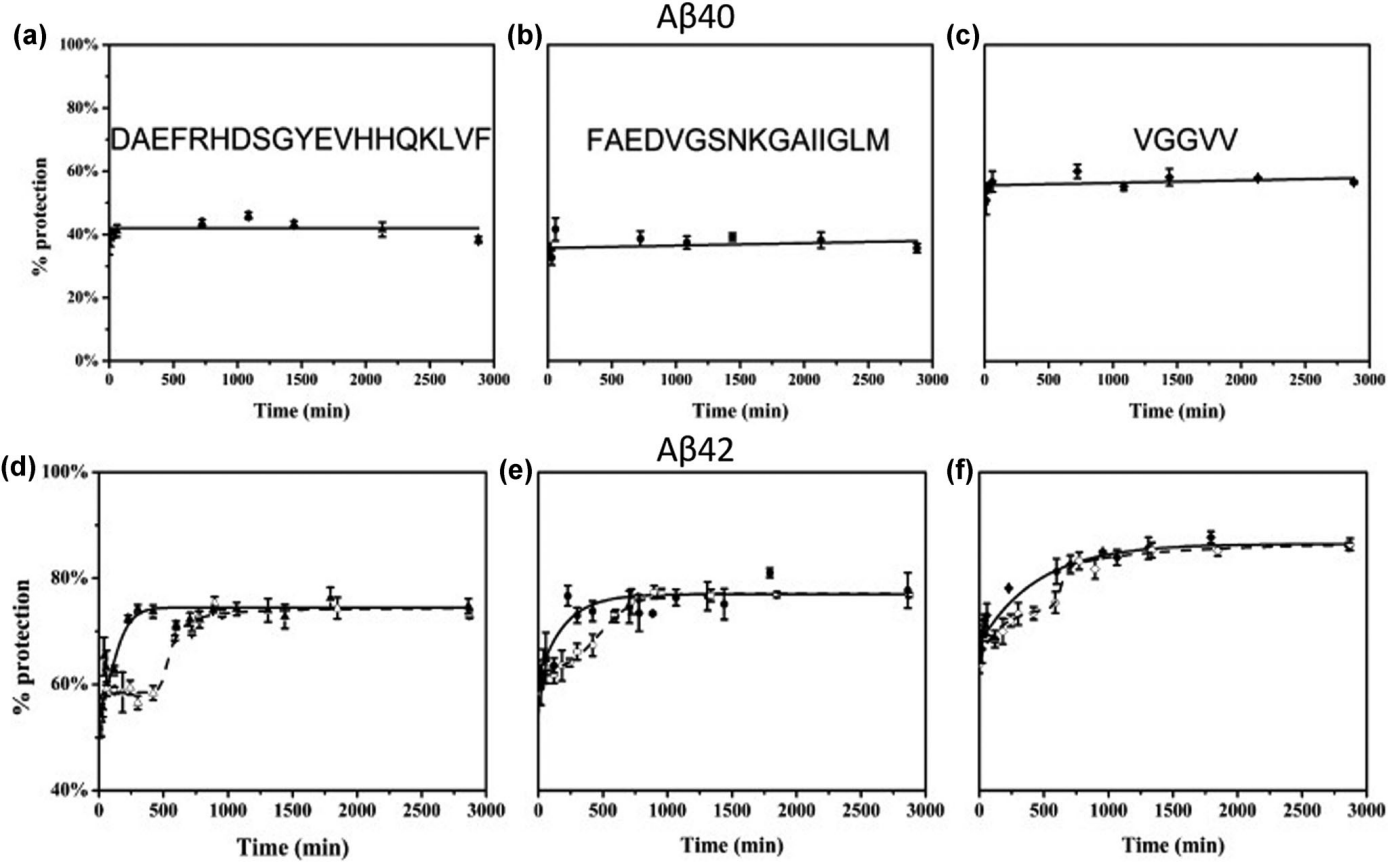
(**a**–**c**) Pulsed HDX results for three peptides from Aβ40. Aggregation was done at 25°C and in the absence of Cu2+. Peptic peptides 1–19 (**a**), 20–35 (**b**), and 36–40 (**c**) are represented by triangles, circles, and diamonds, respectively. (**d**–**f**) Pulsed HDX results for three peptides from Aβ_42_. Aggregation was done at 37°C, with agitation at 150 rpm and in the absence (solid triangles, circles, and diamonds) or presence of Cu^2+^ (hollow triangles, circles, and diamonds). Peptic peptides 1–19 (**d**), 20–35 (**e**), and 36–42 (**f**) are represented by triangles, circles, and diamonds, respectively. Featured in *PNAS* reference Zhang et al. (2013). HDX, hydrogen deuterium exchange.

Pulsed HDX has been used to investigate amyloid β (Aβ) fibrils and oligomers, the formation of which is relevant to Alzheimer's disease (Carulla et al., 2010; Pan, Han, Borchers, & Konermann, 2011; Khanal et al., 2012). These earlier investigations were conducted using a top‐down approach and proposed that Aβ oligomers share similar secondary structure features (i.e. β‐turn‐β) with mature Aβ fibrils. To better understand Aβ aggregation, specifically, at the peptide level, Gross and coworkers employed a novel pulsed HDX workflow followed by pepsin proteolysis that was used for the MS‐based time‐dependent study of aggregation of Aβ_40_ and Aβ_42_ peptides (Zhang et al., 2013). In contrast to normal pulsed HDX, this approach consisted of one rapid (1 min) exchange event referred to as the “pulse” that occurred on a faster timescale compared with the protein folding incubation times. By varying the folding incubation times, the aggregation of the Aβ peptides were followed as a function of time. Comparison of Aβ_40_ and Aβ_42_ showed that the Aβ_40_ peptide had no significant change in deuterium exchange, in contrast, the pulsed HDX of Aβ_42_ produced a sigmoidal shaped curve (Fig. 7). The sigmoidal curve consists of three regimes (1) an initial increase in protection attributed to formation of small Aβ_42_ oligomers, (2) a plateau where there is insignificant conformational change, (3) as larger oligomers form there is no increase in protection, however, these oligomers eventually undergo an autocatalytic reorganization yielding a structure with higher protection. These findings were in agreement with previous studies on amyloid proteins that suggested aggregation occurred by a three‐step process consisting of nucleation, growth, and stabilization (Morris et al., 2008). Using this approach, it was also possible to probe conditions that affect oligomerization of Aβ_42_ such as temperature, agitation, and the presence of metals. These experiments demonstrated that Aβ_42_ aggregation occurs faster at 37°C with agitation than at 25°C, whereas the presence of Cu^2+^ slowed aggregation. The pulsed HDX data was then fitted using the Finke–Watzky model, which assumes aggregation is initiated with a slow continuous nucleation followed by fast self‐catalyzed growth. From the model, the half‐life, *t*_1/2_, could be determined in a site resolved manner. Analysis of the *t*_1/2_ times show that the middle region of the Aβ_42_ peptide quickly undergoes oligomerization (*t*_1/2_ = 1070 ± 30 min) and is therefore the location of initial aggregation. Aggregation then occurs at the hydrophobic C‐terminus (*t*_1/2_ = 1230 ± 30 min) and finally at the hydrophilic N‐terminus (*t*_1/2_ = 1420 ± 20 min). The use of this pulsed HDX method provided novel kinetic information at the peptide level on Aβ_42_ aggregation. This workflow was also extended to interrogate the oligomerization and aggregation of other amyloid forming proteins under various experimental conditions. Adopting the approach to study α‐Synuclein showed (1) that aggregation is concentrated in the core (2) the N‐termini's contribution to aggregation is moderate, and (3) the C‐terminus remains solvent‐accessible throughout the aggregation (Illes‐Toth et al., 2018).

### F FPOP

Hydroxyl radical protein footprinting (HRPF), coupled with MS, imparts an irreversible modification on the solvent exposed side chains of proteins. HRPF can label 19 of the 20 common amino acids in a variety of conditions making it well suited for characterizing proteins structure and dynamics (Xu & Chance, 2005). Chance and coworkers were the first to couple HRPF with MS and demonstrate its utility in studying HOS (Maleknia, Brenowitz, & Chance, 1999; Maleknia et al., 2001; Xu & Chance, 2005). HRPF is complementary to HDX in that HDX examines HOS of proteins by evaluating hydrogen bond stability and protection of the backbone while HRPF exploits radical ion interactions with the solvent exposed side chains to uncover the tertiary structure.

#### i Development of FPOP

**FIGURE 8:**
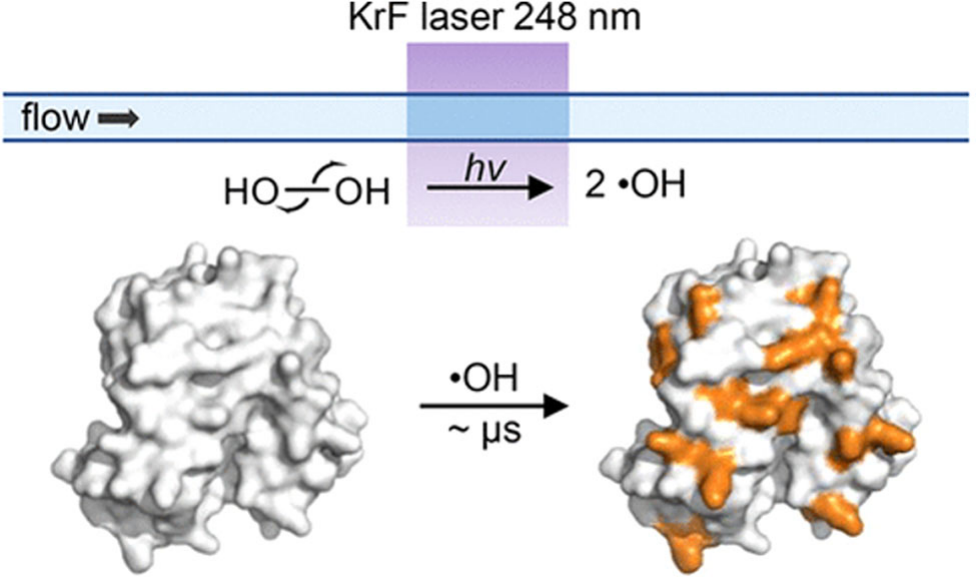
Labeling mechanism of FPOP. (Li, Shi, Gross, 2018. Mass spectrometry‐based fast photochemical oxidation of proteins (FPOP) for higher order structure characterization. Acc Chem Res 51(3):736–744.) FPOP, fast photochemical oxidation of proteins. [Color figure can be viewed at wileyonlinelibrary.com]

Synchrotron‐based HRPF was an important addition to structural proteomics in that it provided medium resolution information on side chain solvent exposure of proteins (Xu & Chance, 2007). However, the use of a synchrotron to generate hydroxyl radicals is not routinely feasible as access to synchrotrons are limited. FPOP provided a more accessible means to generate hydroxyl radicals. In FPOP, hydroxyl radicals are generated using a KrF excimer laser at 248 nm to induce photolysis of H_2_O_2_ in a micro‐flow system (Fig. 8) (Hambly & Gross, 2005).

The first step in implementing FPOP was to optimize the conditions for protein modifications. First, 248 nM was established as the preferred wavelength for H_2_O_2_ photolysis since biologically relevant molecules do not absorb in this region and it is still in the range of H_2_O_2_ absorption (Hambly & Gross, 2005). An issue that needed to be addressed was over oxidation of the protein, as this could lead to unfolding. In response, low H_2_O_2_ concentrations were implemented and glutamine was included as a radical scavenger to minimize the radical lifetime. On the basis of the activity of glutamine with hydroxyl radicals, the time scale of the FPOP reaction was determined to be ~1 μs.

**FIGURE 9:**
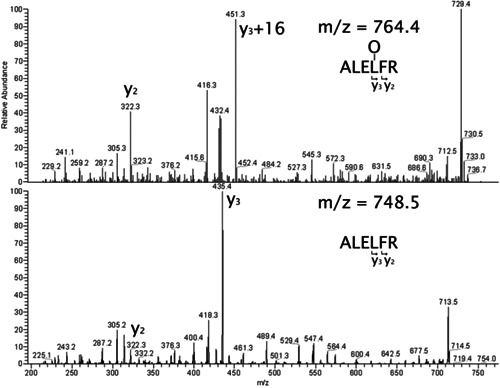
The top image shows MSMS fragmentation pattern of [M + H] oxidized ALELFR. The bottom image displays MSMS fragmentation pattern of [M + H] of *m*/*z* 748.5, The modification is observed on the second leucine. The ion of *m*/*z* 713 or 729 probably formed by loss of water as a result of wide‐band activation followed by loss of ammonia (Hambly & Gross, 2005).

Preliminary FPOP experiments were performed on apomyoglobin and analyzed via bottom‐up LC‐MS/MS sequencing to identify which amino acid residues were modified (Hambly & Gross, 2005). The solvent exposure on modified residues was examined by calculating the solvent accessible surface area (SASA) on the crystal structure of holomyoglobin. For the modified peptides, the modified residue was able to be identified for all but one peptide. A comparison of the SASA with highly reactive residues tryptophan, tyrosine, methionine, phenylalanine, and histidine found these residues can be oxidized even when their solvent accessibility is low. Lower reactive residues such as leucine and isoleucine were shown to be a better probe for solvent accessibility. The preferential modification of leucine in a peptide that also contains a highly reactive phenylalanine residue (Fig. 9) indicates the ability of FPOP to modify solvent accessible residues. Further FPOP studies comparing apo‐ and holomyoglobin revealed variances in oxidation between the two states. This study identified a hinge region on the H‐helix which enabled the binding pocket to close when the protein is in its apo form verifying their earlier results on apomyoglobin (Hambly & Gross, 2007). These studies on myoglobin demonstrated the capacity of FPOP to examine the conformational dynamics of proteins.

**FIGURE 10:**
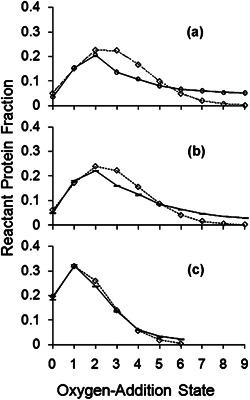
A nonlinear regression best‐fit Poisson distribution was determined. The diamonds along a dotted line show the Poisson distribution. Shown with standard error bars along a solid line, are the averages of the normalized ion counts of four replicates (cases **b** and **c**); case a is singlicate. Plot a is for sample FPOP‐treated without glutamine radical scavenger, post‐FPOP catalase, or post‐FPOP methionine. Plot (**b**) is for sample FPOP‐treated without glutamine. Plot (**c**) is for sample FPOP‐treated with proper controls (Gau et al., 2009). FPOP, fast photochemical oxidation of proteins.

An advantage of the laser‐based method to generate hydroxyl radicals is the fast labeling times. To demonstrate FPOP was labeling on the microsecond timescale, studies on three oxidation sensitive proteins were subjected to FPOP. Charge state spectrum mathematical models were then applied to analyze the data (Gau et al., 2009). To test for conformational changes induced by the time scale of FPOP, they determined the distribution of products resulting from hydroxyl radical labeling and its best fitting Poisson distribution. In this context, a Poisson distribution assesses the independent probability of the hydroxyl radicals substantially changing one or more oxidation targets, which would be reflected in the variance in solvent accessibility across the experimental conditions. The distribution of oxidation states displayed a Poisson curve in the presence of a scavenger and proper controls (Fig. 10c), suggesting that the hydroxyl radicals produced in FPOP could be statistically predicted to occur on a microsecond time scale. This also implies that protein structure is unaffected by the time scale of FPOP. Samples without proper controls are displayed in Figure 10a and b and do not show a Poisson distribution. It was concluded that FPOP, with proper controls, can modify proteins without disrupting its conformation and the data acquired from FPOP experiments reflect native conformations (Gau et al., 2009). The fact that the Poisson distribution fit was not very good for samples that did not include a radical scavenger or a catalase quench indicated the importance of the addition of these experimental controls for studying the native structure of proteins.

#### ii Applications of FPOP

##### ii.1 FPOP for epitope mapping

**FIGURE 11:**
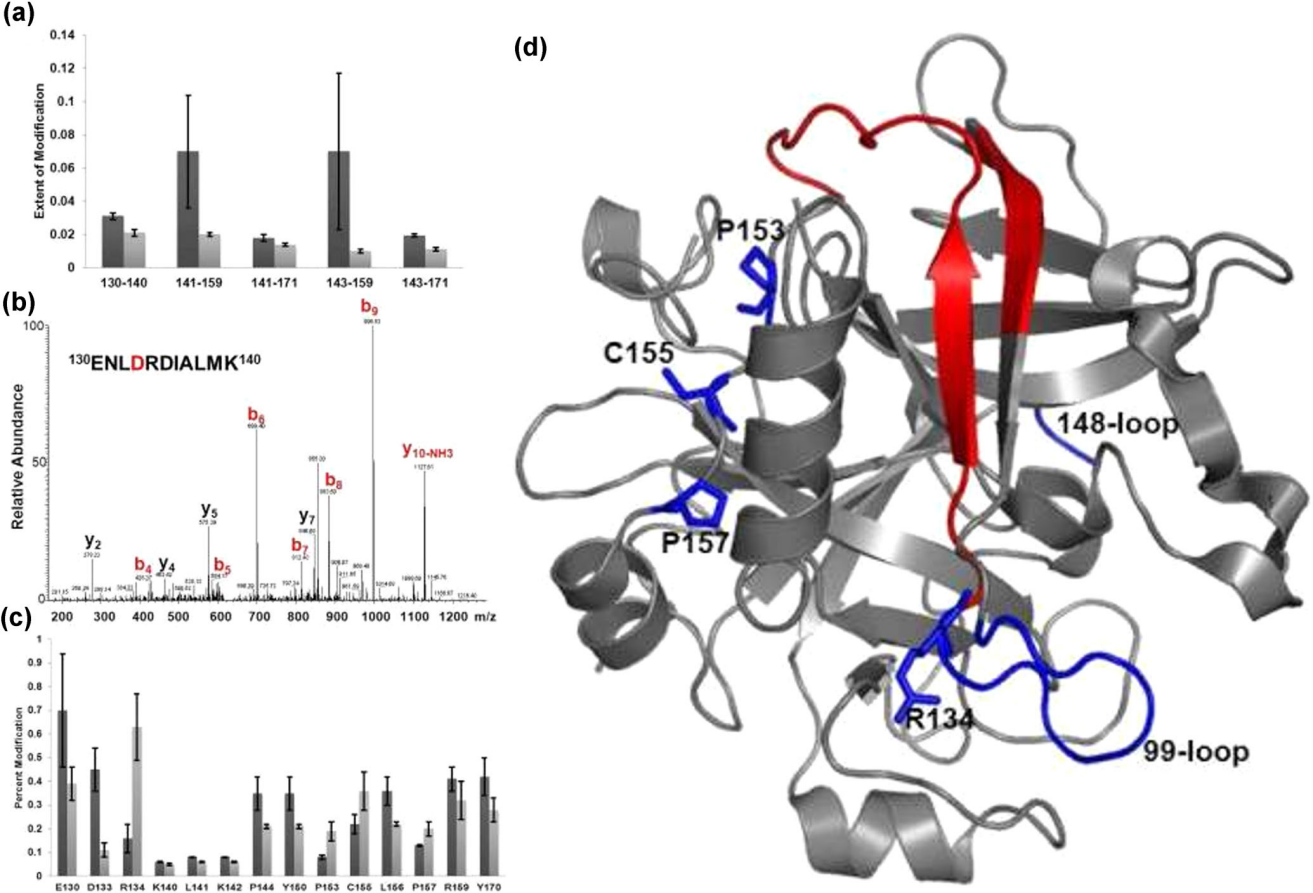
(**a**) Extent of modification of thrombin alone (darker bars) and antibody‐bound thrombin (lighter bars) for five peptides spanning over residues 130–171. (**b**) Product‐ion (MS/MS) spectrum of peptide 130–140, with the fragment ions that contain the labeled residue highlighted in red. (**c**) Extent of modification of thrombin alone (darker bars) compared with antibody‐bound thrombin (lighter bars) at the residue level. (**d**) Structural model of thrombin (pdb file 2AFQ^42^) with the proposed epitope colored red and the loop regions colored blue. The individual residues that show increased modification in antibody‐bound thrombin are specified (Jones et al., 2011). [Color figure can be viewed at wileyonlinelibrary.com]

As the development of new monoclonal antibody (mAb)‐based drugs increases, so does the need for structural characterization for these molecules (Wang et al., 2018). Specifically, the epitope, where the antibody recognizes the antigen, must be defined with special emphasis on side chains as they are major contributors to epitope recognition. Methods like peptide scanning and site directed mutagenesis have proven useful in mapping the epitopes of mAbs but, face limitations especially in determining conformational (discontinuous) epitopes. In response to this, the Gross lab established the use of FPOP for epitope mapping (Jones et al., 2011). The ability of the method to probe the side chain of amino acids makes it especially useful for studying antigen binding. They successfully characterized the epitope of the serine protease thrombin by oxidatively modifying thrombin in both the presence and absence of mAb. Peptide‐level analysis showed decreases in modification for the antibody‐antigen complex on peptide 114–119 indicating this region is at or near the mAb binding site. Decreases in modification were also observed for five tryptic peptides that span 41 amino acids (130–171), surpassing the predicted size of the interaction site (Fig. 11a). To further define the binding site, product ion‐spectra for these peptides were analyzed to extract residue‐level information (Fig. 11b). Quantification on the residue‐level revealed that the interaction site was localized between residues 133–150, a more defined interaction site (Fig. 11c). This work demonstrated the advantage of residue‐level data for higher resolution structural information. The epitope identified by FPOP correlated well to a previous HDX‐MS study (Baerga‐Ortiz et al., 2002). Increases in oxidative modification in the antibody‐antigen complex were also observed in peptides corresponding to the 99‐ and 148‐loop regions of thrombin (Fig. 11d). These changes were attributed to allosteric conformational changes that occur upon antibody binding. These changes were not observed in the HDX‐MS study presumably because they consisted of side chain rotations and HDX‐MS interrogates backbone dynamics (Jones et al., 2011). This work demonstrated that using the two footprinting methods in tandem can provide additional structural information.

To further test the efficacy of FPOP as a structural method for epitope mapping, the Gross lab employed its use for the binding of Adnectin‐1, an mAb mimetic, to human epidermal growth factor receptor (EGFR) (Yan et al., 2014). Owing to EGFR mediation of cell proliferation, migration, and angiogenesis via intracellular signaling that is often dysregulated in cancer, this protein is a potential target for cancer therapeutics. These potential therapeutics include mAb‐based blocking agents that target the extracellular domain of EGFR (exEGFR). Yan et al. studied the interactions site of Adnectin‐1 with exEGFR using modified FPOP conditions. Traditionally for FPOP, glutamine was used as a radical scavenger. On the basis of the concerns of high concentrations of glutamine leading to nonnative conformational changes, histidine was used as a scavenger instead. The high reactivity of histidine with hydroxyl radicals required much lower concentrations, 350 μM, of this amino acid compared with glutamine (15 mM). In addition, histidine is a common component of protein therapeutic formulations demonstrating the utility of FPOP for studying proteins in relevant buffer systems. Statistically significant modification reductions in the Adnectin‐1‐bound EGFR were observed for 14–29 and 57–74 suggesting that the binding site is at or near those locations. Residue‐level analysis localized these decreases in modification to residues L14, L17, F20, F24, and L69. A comparison to the crystal structure of Adnectin‐1 bound EGFR revealed that L17 on EGFR directly interacts with K79 on Adnectin‐1 while L14 and L69 are also involved with interactions at the Adnectin‐1 interface. Although F20 does not directly interact with Adnectin‐1, it is at the buried surface of the binding pocket. F24 is not near the interaction site and decreases in its modification may be due to allosterically controlled side chain orientation or solvent accessibility changes. Furthermore, when the residue level‐FPOP data was compared against HDX‐MS coupled with ETD data, the data was consistent with K13 and Q16 showing decreases in labeling in the complex in the HDX‐MS data. This study further demonstrated the advantages of using for FPOP for epitope mapping as well as the ability to use FPOP in conjunction with other structural methods to obtain orthogonal information on specific interaction sites.

##### ii.2 Temperature jump (T‐jump) and FPOP to investigate protein folding

**FIGURE 12:**
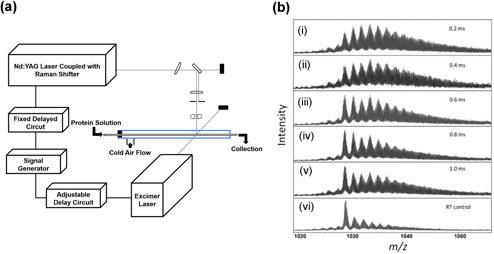
(**a**) Schematic of the T‐jump FPOP flow system intersected by two laser beams at a window in the tube. The time between the two laser pulses is adjustable with the “delay circuit” (**b**). (**a**–**e**) Representative mass spectra of the barstar post‐FPOP as a function of the time between the heating pulse and the FPOP probe. (**f**) Mass spectrum of the barstar post‐FPOP at room temperature as a control Chen et al., 2010. FPOP, fast photochemical oxidation of proteins; T‐jump, temperature jump.

Protein folding studies are essential for understanding protein function. The rapid nature of protein folding puts it out of the regime of many structural methods but the microsecond labeling of FPOP makes it well‐suited for folding studies. To test the efficacy of FPOP for protein folding dynamics studies, the Gross lab integrated FPOP with a T‐jump to probe the folding mechanism of barstar (Chen, Rempel, Gross, 2010). Barstar, a single domain protein that has been extensively used as a model system for protein folding studies, is unfolded at 0°C and folds with increasing temperatures. For these experiments, a two‐laser approach was used (Fig. 12a). The first laser was the standard KrF excimer laser used for FPOP, while the second was a Nd:YAG laser that produced a 1900 nm pulse used for initiating the T‐jump (~20°C). A delay switch was integrated between the two lasers so that FPOP labeling could occur at different times after the T‐jump to monitor protein folding. MS analysis of the intact protein revealed that the extent of oxidation decreased as the protein folded over time (Fig. 12b). A higher number of modifications were observed than in a typical FPOP experiment indicating the protein is in a denatured state even up to 1 ms after the T‐jump. A fit of the centroid mass shift determined a rate constant of 1.5 ms^−1^ which is comparable to the rate constant determined by a previous fluorescence‐based study. This study confirmed that FPOP, combined with T‐jump, is capable of interrogating sub millisecond protein folding kinetics on a global level.

**FIGURE 13:**
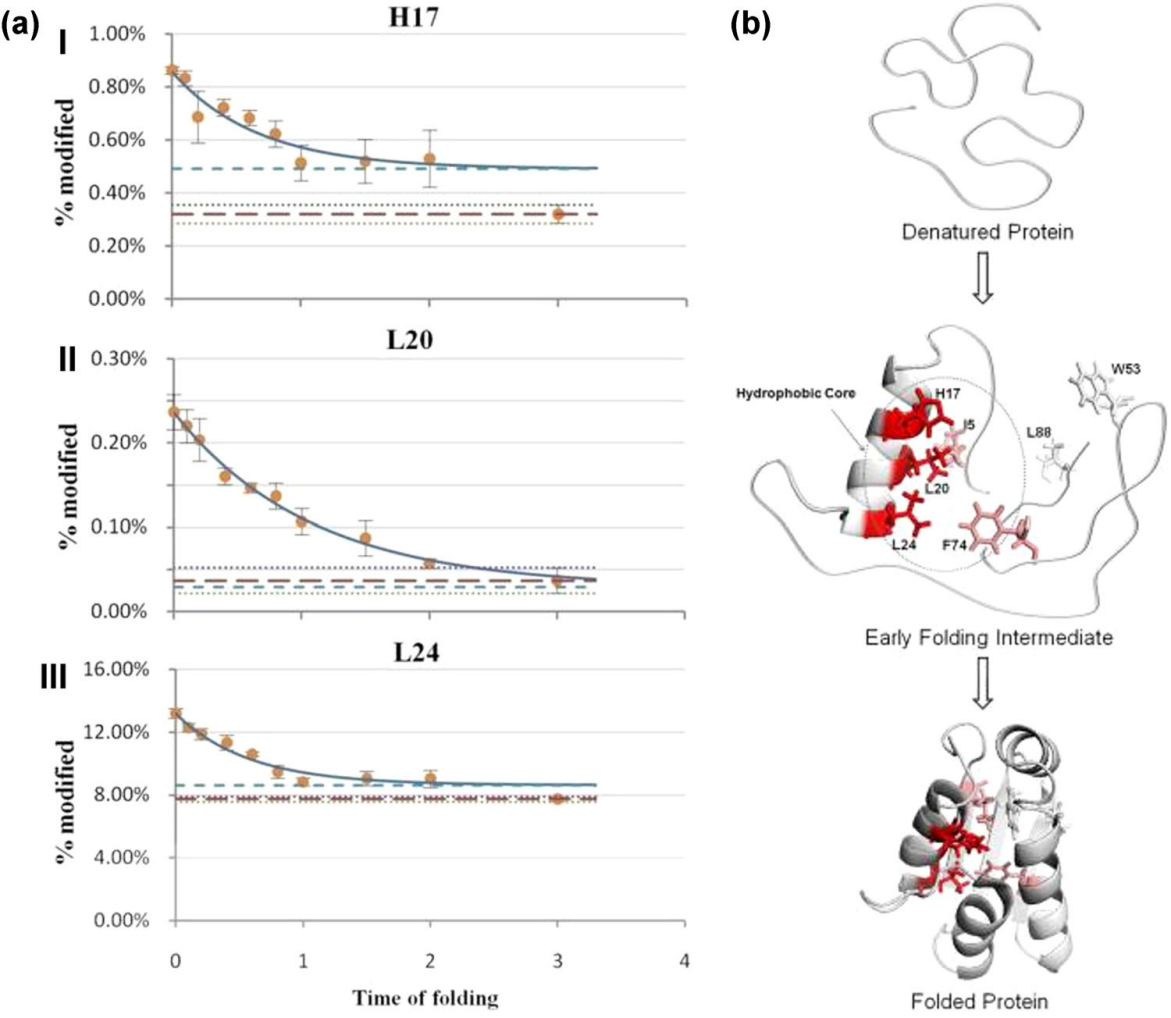
(**a**) Example residues showing significant difference in modification extent between the native and unfolded states. Modification decreases over time (protection increases) is correlated with the early folding event. (**b**) Proposed mechanism of barstar early folding. The folding starts with an ensemble of different conformations, then the protein structure moves with‐in 2 ms to an intermediate possessing moderate structure. Residues colored coded in red are closely associated with the hydrophobic center; white pink indicates regions that weakly interact with the core. Residues colored gray do not participate in the formation of the early folding intermediate (Chen et al., 2012). [Color figure can be viewed at wileyonlinelibrary.com]

To further examine the protein folding kinetics of barstar and its earliest folding events, the T‐jump FPOP workflow was combined with bottom‐up proteomics in order to quantify the extent of oxidation on the residue‐level (Chen et al., 2012). FPOP was performed at eight different time points between 0.1 and 2 s after the T‐jump. To obtain the highest sequence coverage, separate digestions with GluC and with trypsin were performed. This aided in identification of modifications in a 32‐residue segment that lacks lysine and arginine residues. Amino acid residues were categorized based on a comparison of their modification between the native, denatured, and intermediate states. A degree‐of‐folding value was calculated for five residues (H17, L20, L24, I5, and F74) involved in the hydrophobic core of the native state of barstar but had different levels of solvent accessibility in the intermediate state (Fig. 13a). In the intermediate state, L20 had the highest degree of folding signifying its role in the formation of a partially solvent‐excluded core. On the basis of their degree of folding, I5 and F74 appears to be involved in weaker long‐range interactions with the hydrophobic core while W53 and L88 are exposed in the intermediate state. The FPOP data correlated well with the nucleation‐condensation model for protein folding where an ensemble of different conformations folds to an intermediate structure within 2 ms (Fig. 13b). The FPOP data identified helix_1_ as the folding nucleus of barstar which is consistent with the NMR structure of the protein. This worked demonstrated that the integration of T‐Jump with FPOP can be used to study early protein folding events and detect transient states (Chen et al., 2012).

#### iii Using FPOP to Monitor Protein Interactions

**FIGURE 14:**
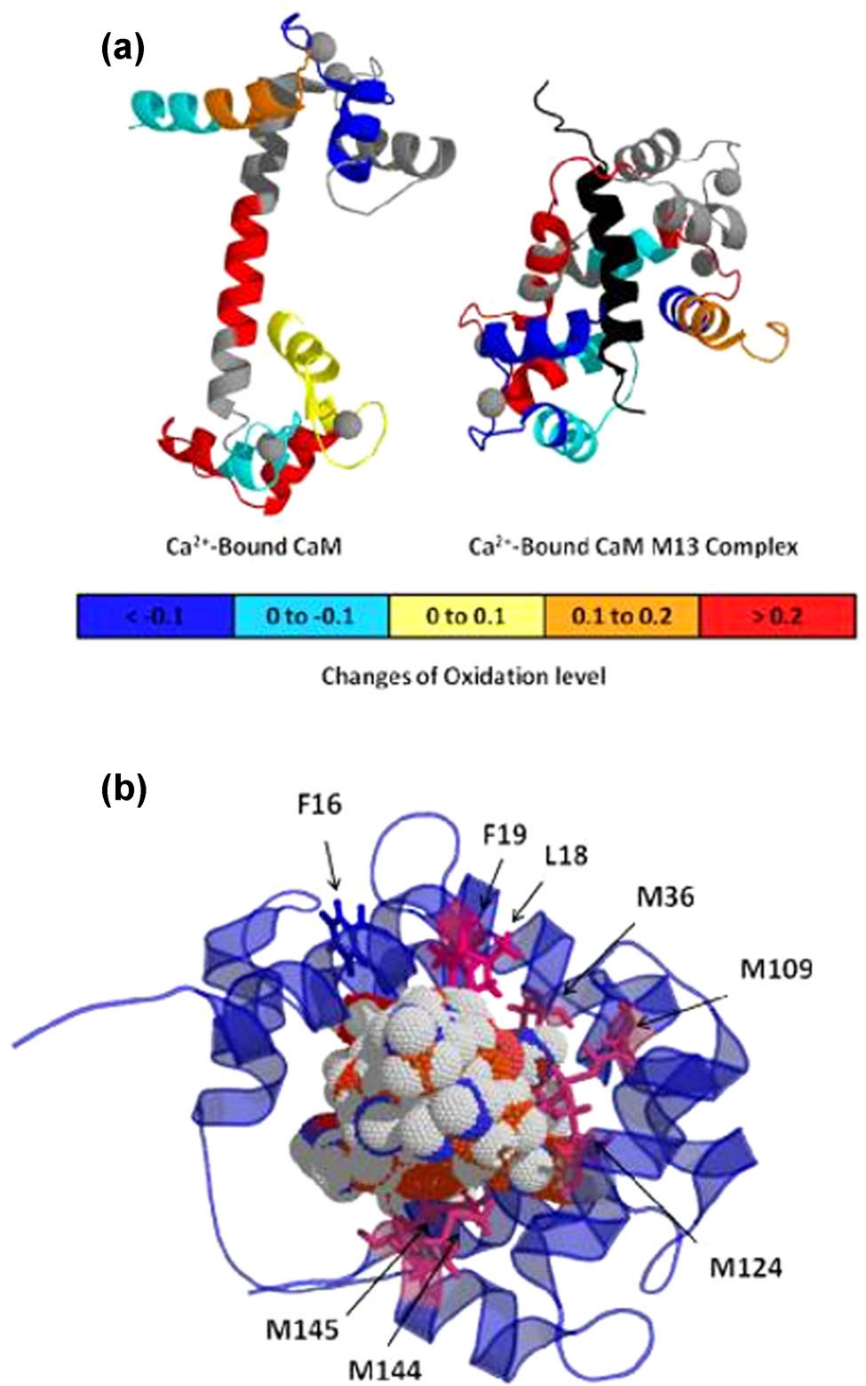
FPOP footprinting results on CaM structures. (**a**) Changes of oxidation are labeled on peptides with different color. M13 is black. (**b**) Bottom view of CaM‐M13 complex. Modified residues (pink) in were detected by an LC‐MS experiment. M13 is in the center of structure with ball shape (Zhang et al., 2011). FPOP, fast photochemical oxidation of proteins; LC‐MS, Liquid chromatography–mass spectrometry. [Color figure can be viewed at wileyonlinelibrary.com]

Characterizing protein–ligand interactions are important to gain insight on biological functions. FPOP is well suited to examine these interactions with residue‐level spatial resolution. In one study, FPOP was employed to compare the conformational effects of peptide ligands bound to calmodulin (CaM). The binding of three different peptide ligands, melittin (Mel), mastoparan (Mas) and M13, the binding domain of skeletal muscle myosin light chain kinase (SK‐MLCK), to both Ca^2+^‐free and Ca^2+^‐bound CaM were studied. A high‐resolution structure is available for CaM‐M13 but not for the other ligands. The goal of this work was to demonstrate whether differences or similarities in the FPOP labeling pattern could be used to inform on whether the binding interactions and binding‐induced structural changes of the other ligands are similar to M13 (Zhang et al., 2011a,b). Differences in the modification between the three complexes at the peptide‐level were examined and the data were separated in to three categories, those with (1) similar modification trends between CaM alone and CaM‐Mel/Mas/13 complexes, (2) varying labeling trends for CaM‐Mel/Mas/M13 complexes than for CaM, and (3) no labeling trend (Fig. 14a). From this comparison, it was determined that peptides 14–21, 22–30, 107–126, and 127–148 are involved with the binding to the peptide ligand or are a result of ligand binding‐induced allosteric conformational changes. Residue‐level analysis further resolves the interaction site by demonstrating that for peptide 14–21 residues L18 and F19 have decreased oxidation in the presence of the peptide ligands while F16 display no change in oxidation indicating this residue may not be involved in direct interactions with the ligand while neighboring residues are involved. With this data they were able to compare the interactions of CaM bound to Mel and Mas with the structure of CaM‐M13 to provide interaction information on these peptides (Fig. 14b). To further determine whether FPOP can be used to compare structures, the spectral‐contrast‐angle was calculated. This approach provides a confidence value (*θ*) that is related to similarity. The *θ* from the comparison of each CaM‐peptide complex is within a range that indicates high similarity, suggesting that each peptide binds to CaM at similar sites and comparable conformational changes. This study demonstrated that FPOP can be used to study protein–ligand interactions. It also showed that the method can be used to determine binding similarities between a series of different ligands when a known reference structure for the protein bound to one of the ligands is available.

#### iv ligand‐titration, fast photochemical oxidation of proteins and mass spectrometry (LITPOMS)‐MS

**FIGURE 15:**
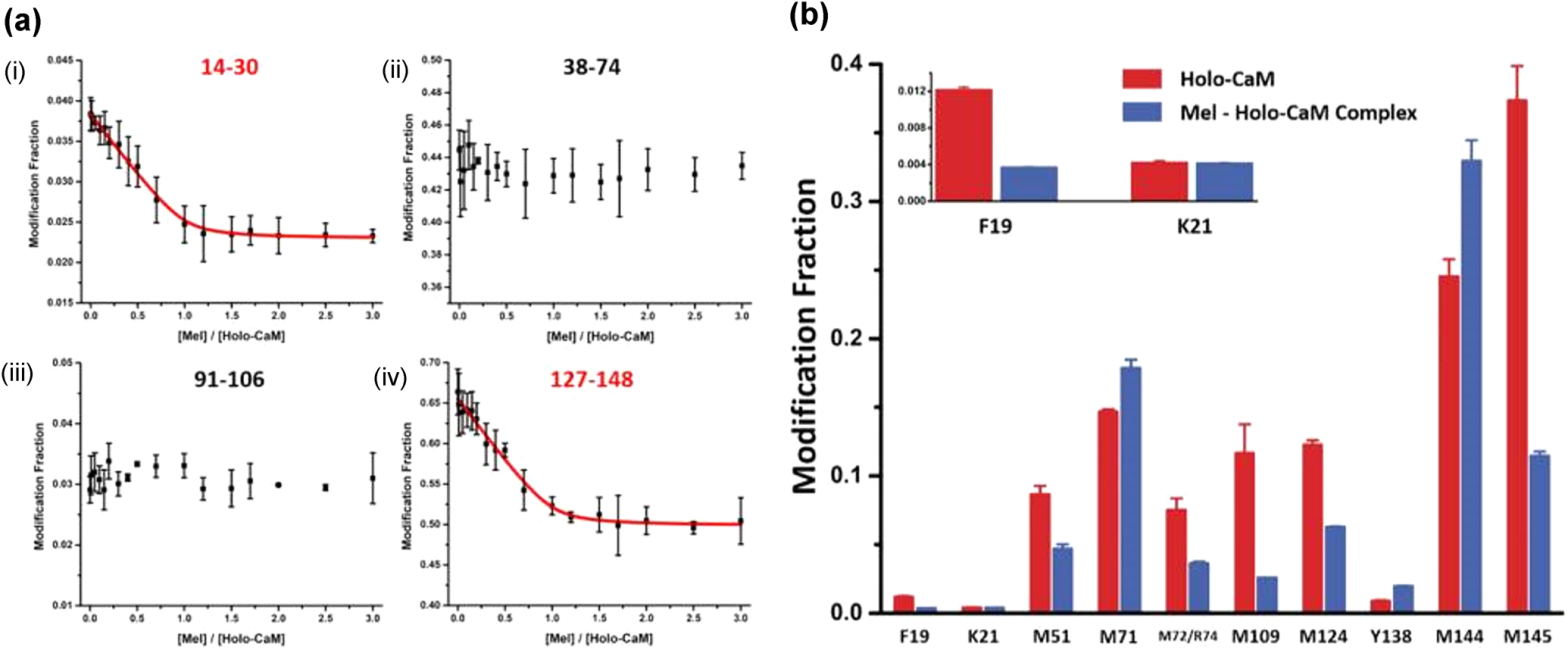
(**a**) Modification fraction as a function of melittin:holo‐calmodulin for selected peptides. Red solid lines in (**a**) and (**d**) are fitting result with the algorithm described previously. (**b**) Modification fraction as a function of melittin:holo‐calmodulin for selected amino‐acid residues. Red bars show modification fraction for holo‐calmodulin itself whereas blue bars represent melittin‐bound holo‐calmodulin where [Mel]/[Holo‐CaM] (Liu et al.,). [Color figure can be viewed at wileyonlinelibrary.com]

More recently, the Gross lab developed a variation of the HDX‐MS‐based PLIMSTEX method for FPOP studies. This method, entitled LITPOMS, can provide binding stoichiometries and binding affinities similar to PLIMSTEX (Liu et al., 2019). However, LITPOMS takes advantage of the fast labeling of FPOP as well as the ability to provide residue‐level structural information to provide increased spatial resolution compared with PLIMSTEX. In the first report of LITPOMS, the binding of melittin (Mel) to calcium‐bound calmodulin (holo‐CaM) was studied. Two concentrations of holo‐CaM provided two different LITPOMS curves for global protein analysis. The higher concentration (10 μM) sample provided a sharp break curve that indicated a 1:1 binding stoichiometry. Peptide‐ and residue‐level analysis were conducted at the lower protein concentration (200 nM) to obtain the binding affinity. Peptides 14–30, 107–126, and 127–148 (Fig. 15a) all showed decreases in labeling indicating they are located at the binding regions. A *K*_d_ of 4.6 nM was calculated based on these peptides, which agrees with a previously recorded *K*_d_ of 3 nM. For residue‐level analysis, F19, M109, M124, and M145 showed decreased in modification upon binding while K21 did not (Fig. 15b). Residues Y138 and M144 showed increased modification upon binding suggesting a binding‐induced conformational change. These data correlate well with a previous cross‐linking study which determine the binding of Mel takes place at the N‐terminus, C‐terminus, and central linker region of CaM.

**FIGURE 16:**
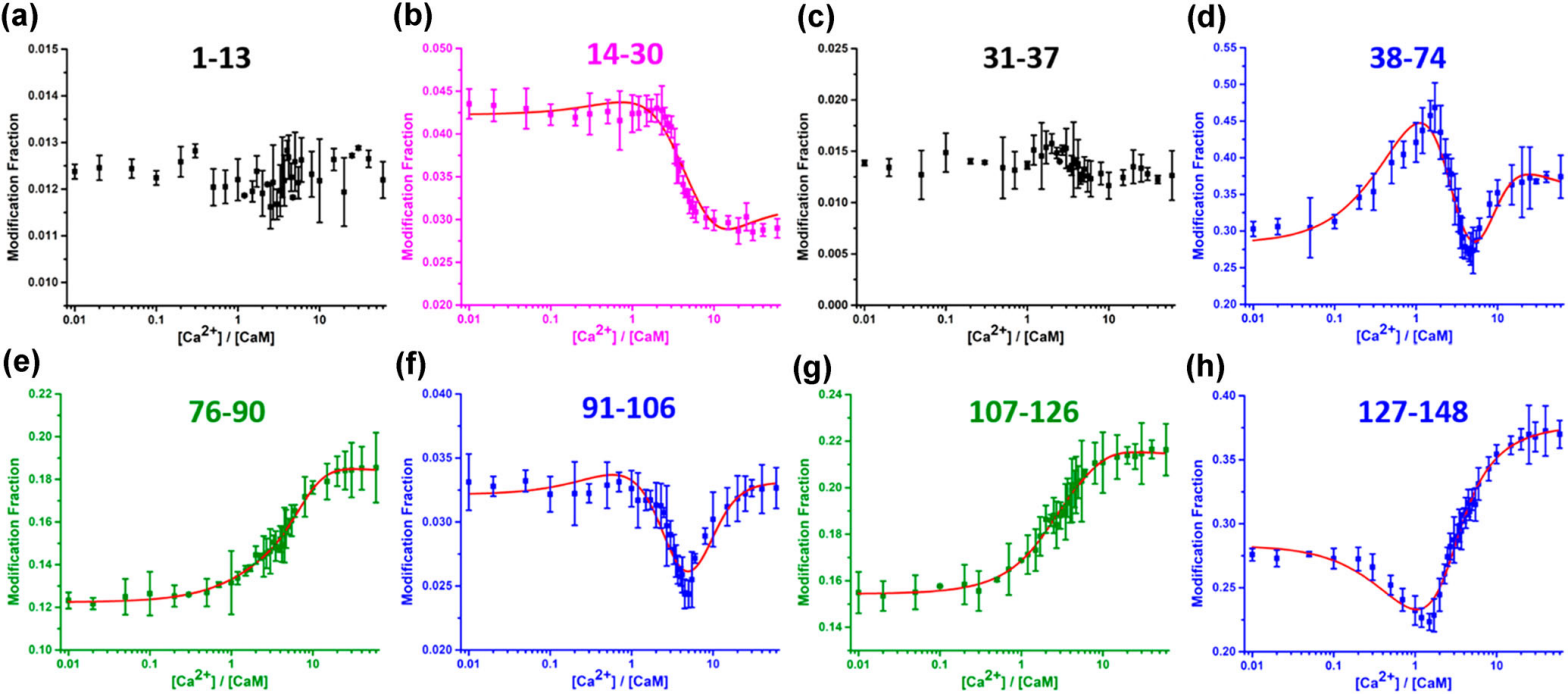
LITPOMS response at peptide level. Modification fractions are plotted as a function of calcium/calmodulin ratio. Four different classes of behaviors are shown in black (**a**, **c**), magenta (**b**), blue (**d**, **f**, **h**), and olive (**e**, **g**). Red solid lines in (**b**, **d**–**h**) are from fitting using an algorithm reported previously. (Liu et al., 2019). LITPOMS, ligand‐titration, fast photochemical oxidation of proteins and mass spectrometry. [Color figure can be viewed at wileyonlinelibrary.com]

The utility of LITPOMS for studying conformational changes and cooperativity in addition to binding affinity of stoichiometry was studied using the CaM‐Ca^2+^ complex. To fully characterize all of the aspects of calcium binding to CaM, it has been necessary to employ multiple techniques. In this study, the Gross group demonstrated that LITPOMS can be used as a single approach to detail conformational changes, order of binding, binding affinities, cooperativity, and allostery. When titrated with calcium, different regions of CaM showed different behaviors that were broken down into four classes (Fig. 16). Peptides 76–90 and 107–126 (Fig. 16e and g) demonstrate a loss of protection with increasing concentrations of calcium indicating these regions becomes more solvent accessible as CaM undergoes conformation changes in response to calcium binding. Peptides 1–13 and 31–37 (Fig. 16a and c) had no significant changes with increasing calcium indicating they are not involved in binding or conformational changes. Peptide 14–30 (Fig. 16b) displays constant labeling early in the titration but is protected from labeling at later concentrations. This points to the cooperativity of calcium binding where this region of the protein only binds to calcium after the [Ca^2+^]/[CaM] = ~2. Peptides 38–74, 91–106, and 127–148 (Fig. 16d, f, and h) report on the conformation changes that occur in the protein with increasing concentrations of calcium demonstrating the allosteric behavior of calcium binding. The LITPOMS titration was also able to determine order of binding for the four calcium binding sites as well as binding affinities for each site. For two of the binding sites, the affinities calculated from LITPOMS were within a factor of 1.6 to literature values. This study demonstrated that LITPOMS can be used as a single approach to interrogate complex protein–ligand interactions including binding affinities, conformational change, cooperativity, and allosteric behavior, with high spatial resolution.

### g Footprinting Innovations

**FIGURE 17:**
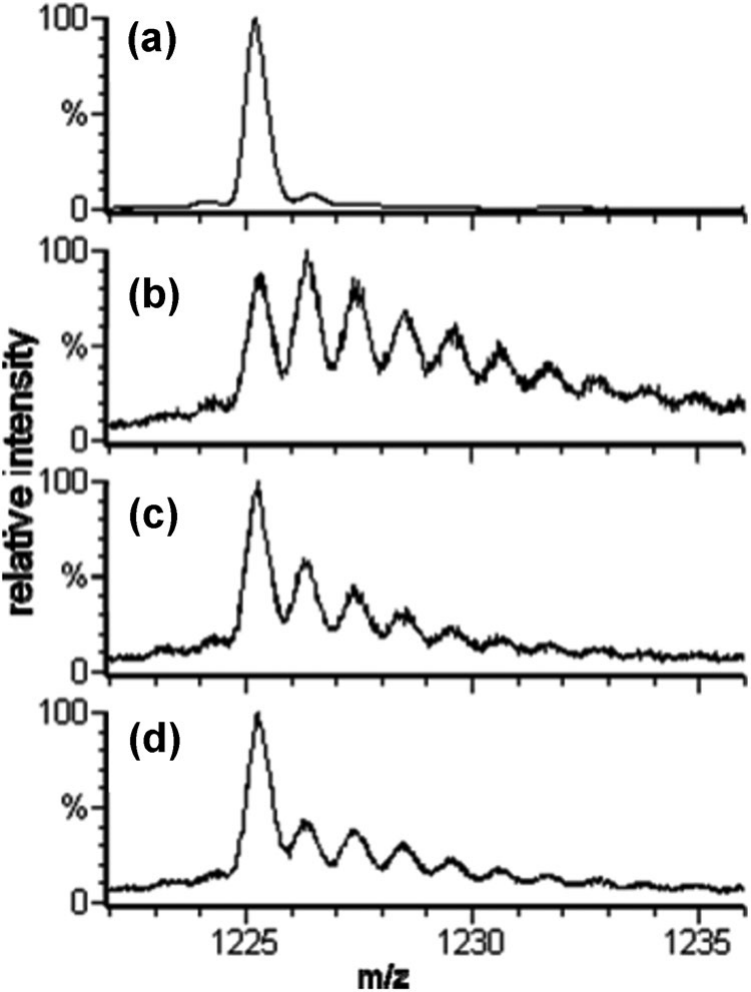
The ESI‐QTOF mass spectra of the 15th charge state of *β*‐lactoglobulin subjected to different labeling conditions. Spectrum (**a**) is of the 15 mM Na_2_S_2_O_8_ control, absent only laser irradiation; (**b**) is of 15 mM Na_2_S_2_O_8_ FPOP; (**c**) is of 5 mM Na_2_S_2_O_8_ FPOP; (**d**) is of 15 mM H_2_O_2_ FPOP. Adapted with permission from reference (Gau et al., 2010). ESI‐QTOF, electrospray ionization‐Q‐TOF, quadrupole time‐of‐flight; FPOP, fast photochemical oxidation of proteins.

To increase the versatility of the FPOP platform, the modifying reagent can be changed in lieu of the hydroxyl radical. One such reagent is the sulfate radical anion (SO_4_^−^•) whose precursor is sodium persulfate. Owing to the sulfate radical anion having a higher reduction potential than the hydroxyl radical it was assumed that it would produce an increased number of oxidative modifications (Gau et al., 2010). The sulfate radical was generated using the standard FPOP setup with sodium persulfate replacing hydrogen peroxide. The modification of β‐lactoglobulin by the sulfate radical anion and the hydroxyl radical were compared. When using the same concentration of the precursor (15 mM), the protein was significantly more modified with the sulfate radical anion (Fig. 17). The concentration of sodium persulfate had to be reduced by threefold to achieve the same level of oxidation as the hydroxyl radical sample because of the higher reduction potential of SO_4_^−^•.

**FIGURE 18:**
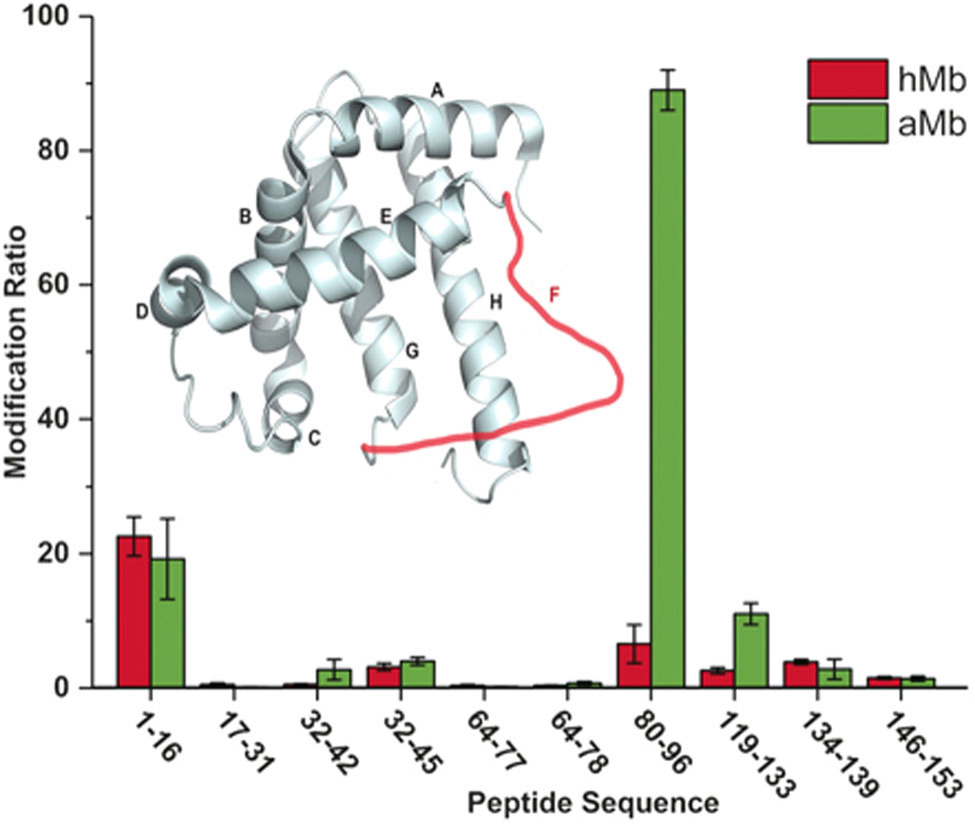
Comparison of modification extents of constituent peptides from hMb and aMb. Significant differences in modification occur in the region represented by peptide 80–96, marked in red. hMb contains a helix region (F helix) in the region of peptide 80–96 whereas aMb doesn't form a F helix. Adapted with permission from reference (Cheng et al., 2017). [Color figure can be viewed at wileyonlinelibrary.com]

**FIGURE 19:**
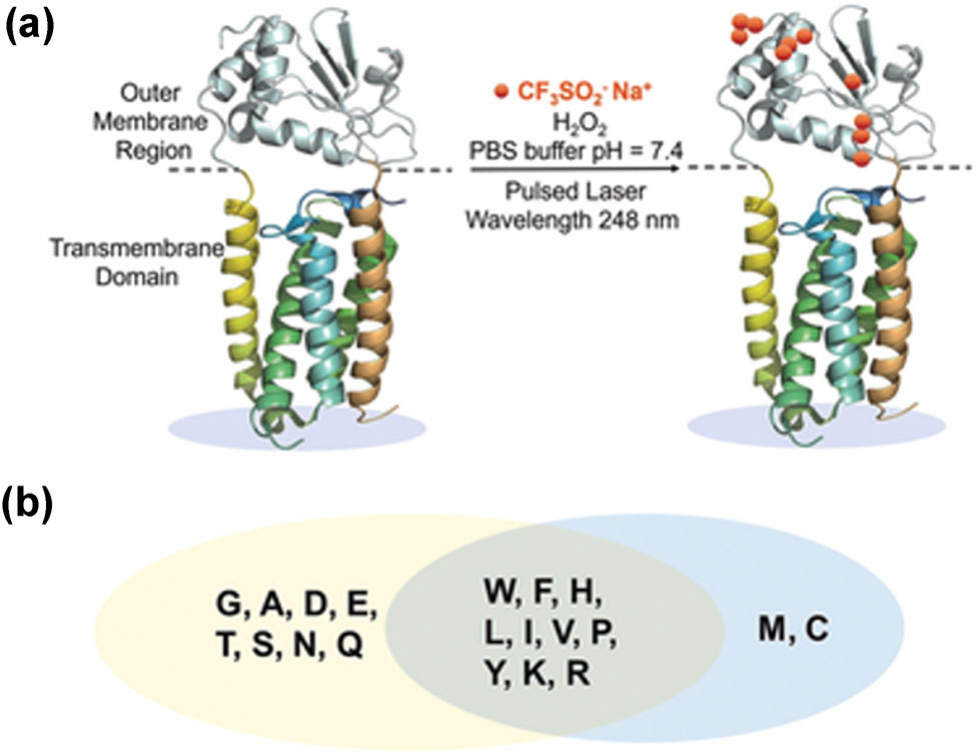
Site reactivity for a native protein higher order structure. (**a**) Scheme for fast trifluoromethylation of VKOR membrane proteins. CF3‐modified sites are in orange. (**b**) Venn diagram comparing •OH‐reactive residues (in blue) with •CF3‐reactive residues (in yellow); residues in overlap are reactive to both OH and CF3 radicals. ·OH‐silent residues are in yellow crescent region. Adapted with permission from reference (Cheng et al., 2017). PBS, phosphate‐buffered saline. [Color figure can be viewed at wileyonlinelibrary.com]

The trifluoromethyl radical, CF_3_•, is another reagent in the footprinting toolbox and can modify 18 of 20 amino acids. The trifluoromethyl radical can modify proteins in the presence of detergent under native conditions implying potential to modify membrane proteins (Cheng et al., 2017). This approach was validated using apo‐ and holo‐forms of myoglobin. The results show that trifluoromethylation does not occur at residues spanning across a helix region (peptide 80–96) found in the holomyglobin but absent in apomyglobin (Fig. 18), indicating that the footprinting reagent is sensitive to the structural differences of the two forms of the protein. To show the utility of this footprinting reagent it was used to modify the membrane protein vitamin K epoxide reductase (VKOR). The results of which illustrate that ten outer membrane residues were modified (Fig. 19a). Also, noteworthy, the trifluoromethyl radical modified residues Gly, Ala, Val, Ile, and Leu on the VKOR protein highlighting that it is active in regions were the hydroxyl radical is not (Fig. 19b).

The footprinting methods that have been covered thus far are general labeling strategies capable of modifying most of the amino acids. Alternatively, an amino acid specific modification reagent can also be employed. Using iodobenzoic acid under the standard FPOP conditions to generate the iodide radical, made it possible to selectively modify histidine and tyrosine (Chen et al., 2012). An advantage of using iodobenzoic acid is that it will not modify the protein unless subjected to laser radiation unlike hydrogen peroxide, therefore, all observed modifications can be attributed solely to the iodine radical. Also, modification by the iodine radical is fast and is likely to occur before unfolding events can occur. The apo‐ and holo‐forms of carbonic anhydrase, a metalloenzyme that catalyzes the hydration of CO_2_, were modified using the iodine radical. Comparison of the extent of modification show no considerable difference which agrees with previous findings that the removal of the zinc prosthetic group does not cause a significant change to the tertiary structure (Brewer et al., 1968; Håkansson et al., 1992).

**FIGURE 20:**
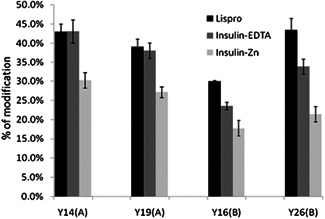
Plot of the yield of iodinated tyrosine residues in lispro, insulin with EDTA and the zinc form of insulin, analyzed by the bottom‐up strategy. Adapted with permission from reference (Chen et al., 2012). EDTA, ethylenediaminetetraacetic acid.

Conversely, the ability of insulin to oligomerize to a hexamer is determined by the presence or absence of a zinc ion. Given this structural dependence on zinc, r‐human insulin with zinc, r‐human insulin‐ethylenediaminetetraacetic acid (EDTA), and lispro insulin were modified via iodination. Under the conditions of the experiment r‐human insulin with zinc is present either as a dimer or hexamer, whereas r‐human insulin‐EDTA, without the stabilization of zinc, is unable to oligomerize to the hexamer form and exists as either monomer or dimer. As discussed earlier, lispro is an insulin analog designed to exist mainly as a monomer. Comparing lispro and insulin‐EDTA reveal that the extent of modification on the A chain, which does not significantly participate in oligomerization, is similar. This contrasts with the modification on the B chain in which lispro was substantially more modified than insulin‐EDTA a finding that is attributed to protection afforded by dimerization of insulin‐EDTA. Similarly, the insulin and zinc sample with its ability to hexamerize had less modification of all modifiable tyrosines (Fig. 20).

**FIGURE 21:**
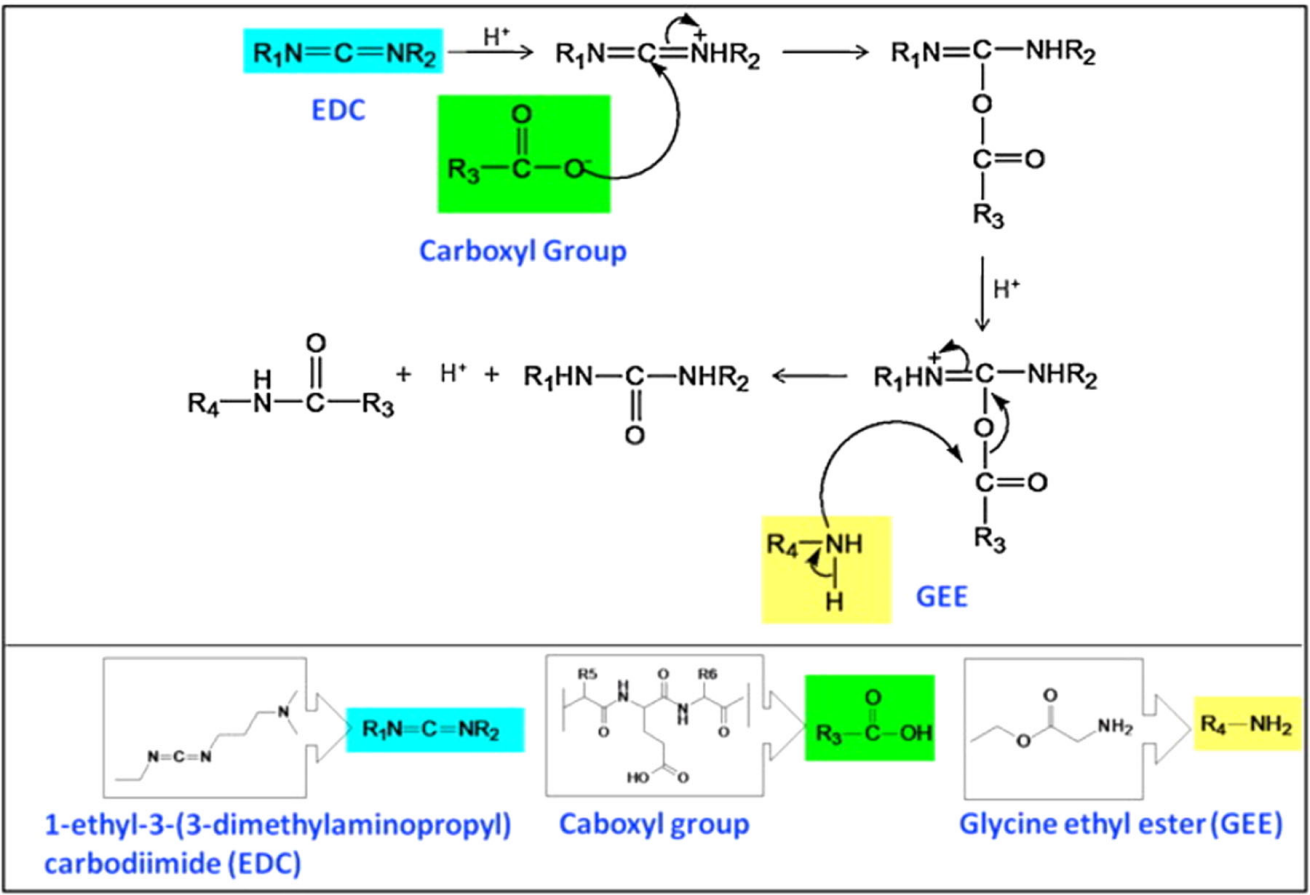
Modification reaction by the GEE coupling reaction. Adapted with permission from reference (Zhang et al., 2012). GEE, glycine ethyl ester. [Color figure can be viewed at wileyonlinelibrary.com]

The Gross lab also explored the irreversible and site‐specific modifications caused by the 1‐ethyl‐3‐(3‐dimethylaminopropyl)‐carbodiimide (EDC)‐mediated coupled reaction between glycine ethyl ester (GEE) and the carboxyl group of solvent accessible glutamate and aspartate residues (Fig. 21) (Zhang et al., 2012). Briefly, EDC activates the carboxyl group of the protein, the activated carboxyl then undergoes nucleophilic attack by GEE as EDC and the oxygen of the original carboxyl act as a leaving group. Additionally, the laser free nature of this footprinting technique makes it suitable to interrogate photosensitive systems. Using this labeling strategy, the architecture of the green sulfur bacteria, *Chlorobaculum tepidum*, photosystem was investigated. The photosystem is composed of the Fenna‐Matthews‐Olson (FMO) protein which acts as a tether to connect the light‐harvesting complex chlorosome to the membrane‐embedded reaction center.

**FIGURE 22:**
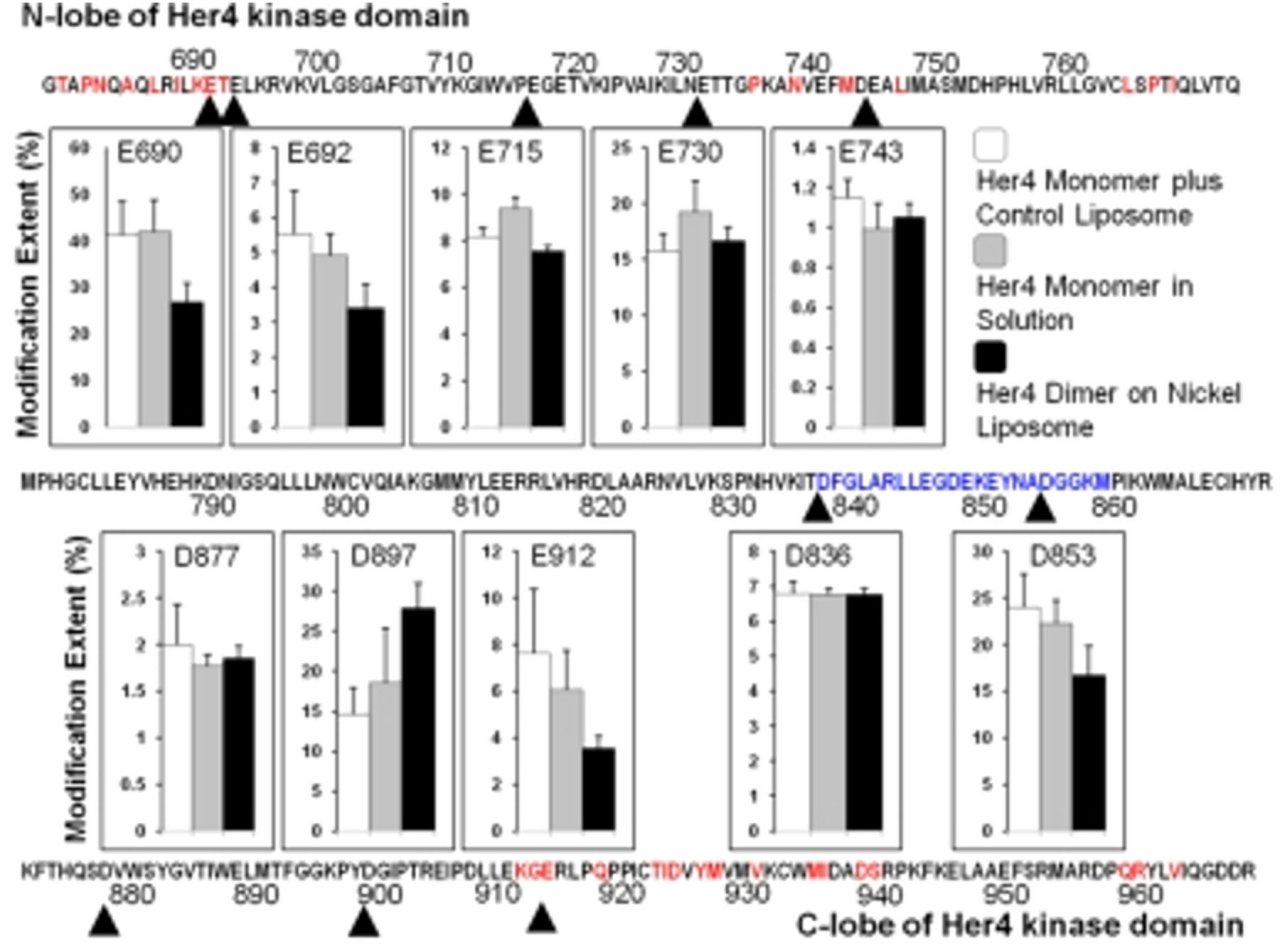
Carboxyl group modification extents for Her4 kinase‐domain protein. Her4 residue numbering is based on the mature, full length protein minus its signal peptide and matches the numbering used in Qiu et al., 2008. The residues involved in dimer interface in crystal structure are labeled in red. The residues from activation loop are labeled in blue. Data represent mean and standard deviation of three independent samples. Adapted with permission from reference Zhang, 2011a. [Color figure can be viewed at wileyonlinelibrary.com]

Using this labeling strategy, only the carboxyl groups of glutamate and aspartate residues were modified resulting in a unique mass shift of +85 and +57 Da (Zhang et al., 2012). In addition to the high specificity, they showed that 79% of possible reactive sites on Her4, a transmembrane receptor tyrosine kinase, were modified (Fig. 22) (Zhang et al., 2011a,b). This validates that GEE footprinting can detect conformational changes and probe conformational variations induced by binding. These types of carboxyl group modifications are complimentary to traditional pre‐existing structural biology tools like HDX, NMR, and FPOP.

Another footprinting approach utilized photoleucine, a photosynthetic protein, to generate carbene to modify proteins resulting in a mass shift of +115. In this work, it was reported that modification via carbene occurs at a nanosecond timescale which is faster than FPOP and also signifies that reaction takes place before unfolding can occur (Do Kwon et al., 2015). Perhaps the most appealing aspect of this form of footprinting is that it reacts with amino acids (Gln, Glu, Asp, Asn, Ala, and Gly) that are not reactive in a typical FPOP experiment. The evolution of FPOP has demonstrated that the FPOP platform can be implemented using a suite of modifying reagents that can be customized to fit the experimental requirements.

### h Combined Protein Footprinting Methods

**FIGURE 23:**
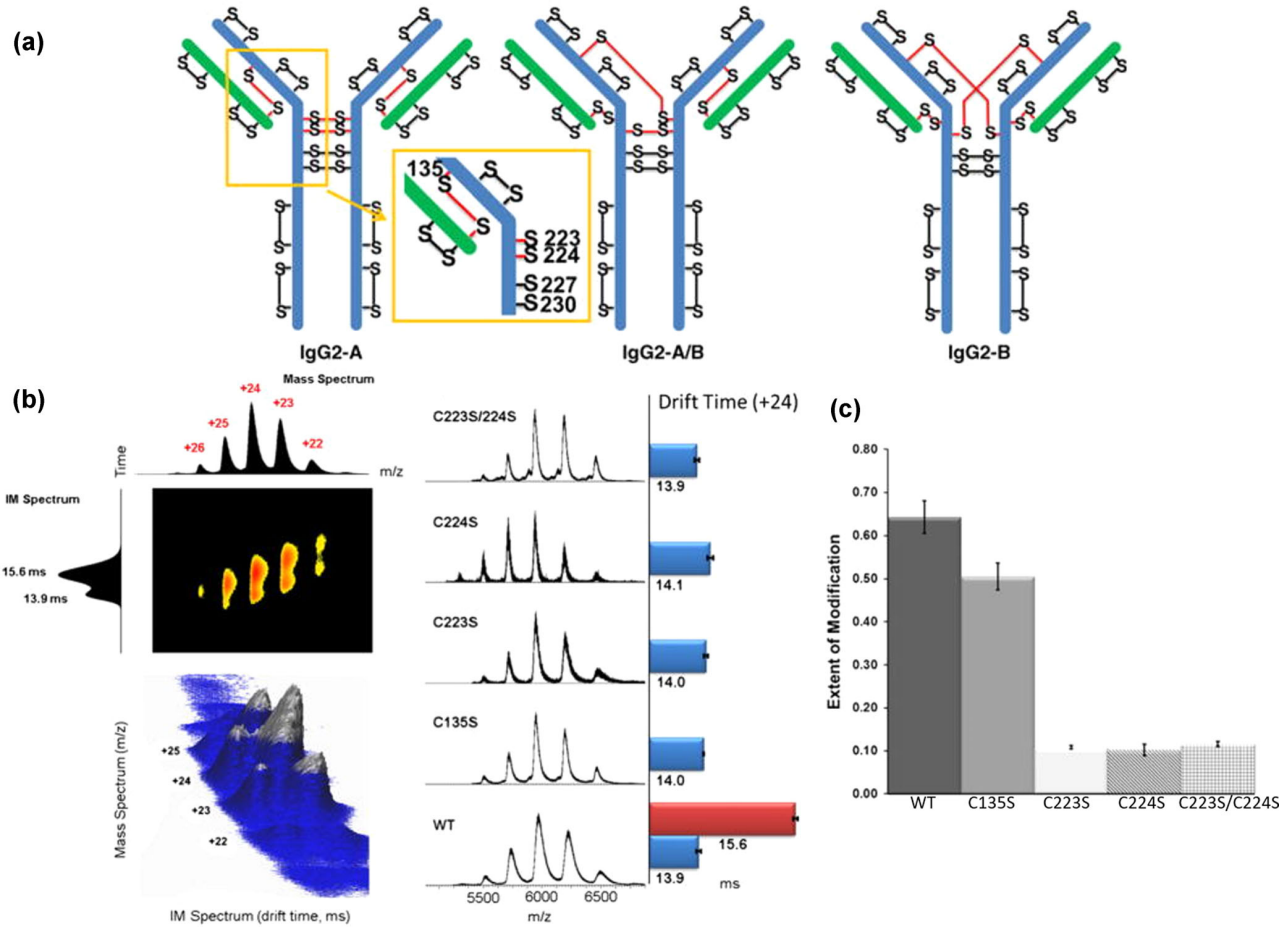
Disulfide isomers of IgG2. IgG2 antibodies have three different disulfide isoforms, IgG2‐A (left), IgG2‐A/B (middle), and IgG2‐B (right). The relevant cysteine residues are labeled in the inset. The disulfide bond between C135 and C214 in IgG2‐A is similar to disulfide bonding pattern in IgG4 antibodies. The disulfide bond between C214 and C223, observed in IgG2‐B, is similar to that of an IgG1 antibody. (>**b**) IM of wild type (WT) and mutants. (shaded red) The spectrum of WT sample, plotted in two dimensions, *m*/*z* and drift time. The IM spectrum of the most abundant charge state was extracted from the 2D spectrum and plotted on the left side to show two conformers. (shaded green) 3D (*m*/*z*, drift time, and ion counts) spectrum of WT sample showing two conformers for +23 and +24 charge states. (shaded blue) Mass and IM spectra of WT and mutants ionized by native ESI afford a narrow charge distribution from m/z 5250 to 6750. The drift times (centroid value) of +24 charge states were plotted on the right. The precision is ~±0.1 ms. (**c**) FPOP labeling of the hinge region. Adapted with permission from reference (Jones et al., 2013). 2D, two‐dimensional; ESI, electrospray ionization; FPOP, fast photochemical oxidation of proteins; IgG, immunoglobulin G. [Color figure can be viewed at wileyonlinelibrary.com]

An additional feature of MS‐based footprinting is that it can be applied in combination with other structural characterization methods. For example, Jones et al. using FPOP combined with ion mobility and top‐down mass spectrometry characterized immunoglobulin G2 (IgG2) mAbs on the basis of their HOS (Jones et al., 2013). To evaluate the sensitivity of the combined approach to HOS, members of IgG2 subclass with isomeric disulfide structures were analyzed (Fig. 23a). Ion mobility experiments showed that the wild type (WT) IgG2 exists in two major isomers and that each of the mutants is more compact than the extended WT isomer (Fig. 23b). Although ion mobility can be performed quickly, the method is limited to global structural observations. This is contrast to top‐down mass spectrometry and FPOP which both provide site‐specific information. In this study, top‐down mass spectrometry reveals that flexibility occurs in the light chain for all mutants, however, it is unable to offer residue‐level resolution. FPOP provided peptide or residue‐level resolution for the entire antibody and was used to localize the conformational variation observed by ion mobility, specifically, identifying the hinge region as the location of these differences (Fig. 23c). Regarding the findings from top‐down analysis, FPOP was able to verify the flexible regions in the light chain. Finally, FPOP modification of the complementarity‐determining region (CDR) of the antigen showed that the region is solvent‐accessible. In this concerted approach, ion mobility and top‐down rapidly provide global to regional behavior which could then be more rigorously explored using the amino acid‐level resolution of FPOP.

Taking advantage of the orthogonal information offered by FPOP and HDX, a combined approach was used to investigate the binding of human bromodomain‐containing protein 4, BRD4, to bind a hydrophobic benzodiazepine inhibitor (Li et al., 2019). HDX studies performed in this investigation revealed that BRD4 was stabilized by binding the inhibitor but could not offer insight into the location of the binding site. In contrast, FPOP experiments unveiled an important binding region in the hydrophobic cavity that was substantiated by X‐ray crystallography studies. The results of this work reinforced the utility of an orthogonal footprinting approach.

**FIGURE 24:**
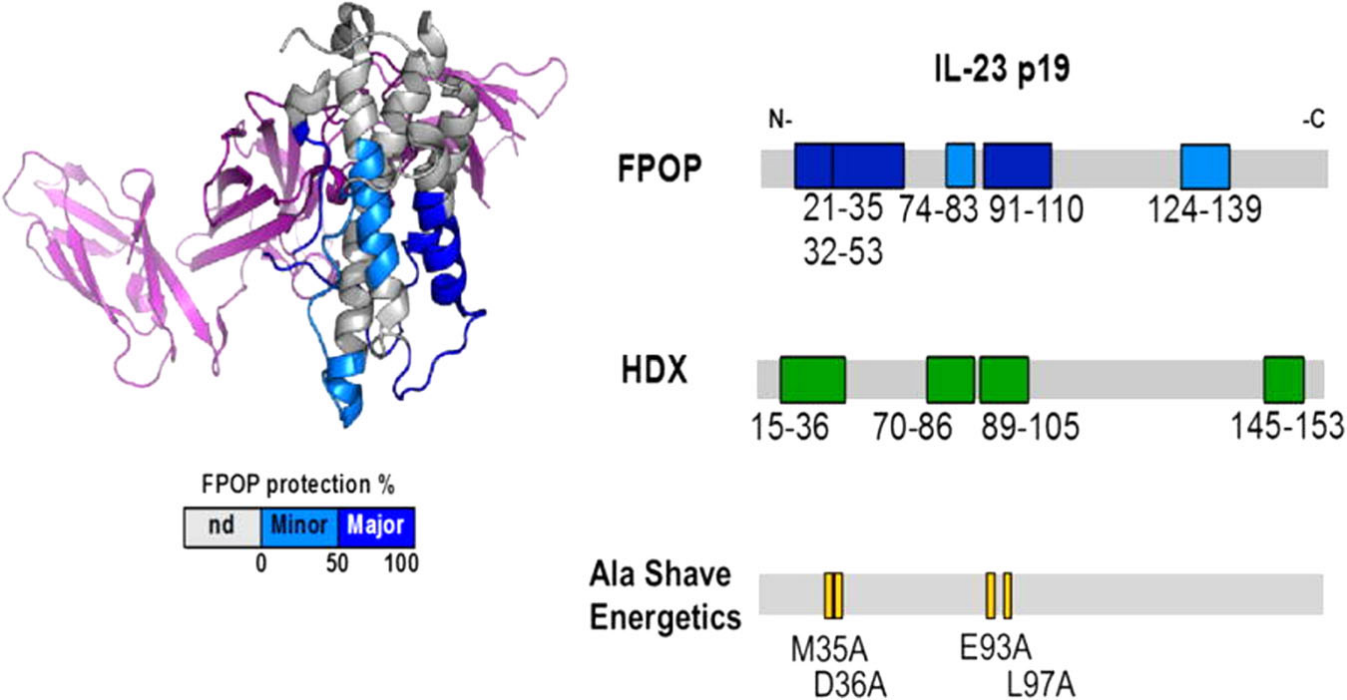
Epitope regions determined by FPOP, HDX, and Ala Shave Energetics as mapped on the linear sequence of the IL‐23 p19. Noteworthy, there are regions that are “silent” in HDX but “active” in FPOP and vice‐versa highlighting the complementary nature of HDX and FPOP. Adapted with permission from reference (Li et al., 2017). FPOP, fast photochemical oxidation of proteins; HDX, hydrogen deuterium exchange; IL‐23, interleukin‐23. [Color figure can be viewed at wileyonlinelibrary.com]

The combined approach was also used to map the energetic epitopes of interleukin‐23 (IL‐23) (Li et al., 2017). This work showed that there are regions that can be explored by both HDX and FPOP but, there are also regions exclusive to each method. For example, in the case of the IL‐23 study, HDX was able to detect peptide binding occurring between residues 143–153 of the protein, a region that did not produce oxidation products as it was unreactive to hydroxyl radicals (Fig. 24). Analysis by FPOP identified a binding site between amino acids 124–139 that was not detected in HDX, this discrepancy was attributed to the fact that HDX operates on a larger timescale than FPOP (sub millisecond) and may be unable to detect changes that occur rapidly. Alanine shave mutagenesis, which probes the energetics of the landscape, was also used in tandem with the MS‐based techniques to distinguish regions that directly participate in epitope binding versus those that are only affected structurally.

## III CONCLUSION

The field of MS‐based protein footprinting has seen significant advancements in recent years that have greatly expanded the utility of these methods. Both general labeling strategies and amino acid specific methods have proven useful in the study of proteins. Combination of footprinting strategies or other MS‐based structural techniques such as ion mobility, top‐down proteomics, and X‐ray crystallography (Niu et al., 2020) have demonstrated the complementary nature of protein footprinting. Michael Gross has been at the forefront of innovation in the field. His lab has provided a significant body of work that has contributed to the foundations of the field.

## ABBREVIATIONS

Aβ: amyloid β
CaM: calmodulin
CCS: collisional cross‐section
CDR: complementarity‐determining region
dPLIMSTEX: dilution strategy protein–ligand interactions in solution by MS, titration, and, H/D exchange
EDC: 1‐Ethyl‐3‐(3‐dimethylaminopropyl) carbodiimide
EGFR: epidermal growth factor receptor
ESI: electrospray ionization
exEGFR: extracellular domain of epidermal growth factor receptor
FMO: Fenna‐Matthews‐Olson
FPOP: fast photochemical oxidation of proteins
GEE: glycine ethyl ester
HDX: hydrogen deuterium exchange
HOS: higher order structure
HRPF: hydroxyl radical protein footprinting
IMS: ion mobility spectrometry
LC‐MS/MS: liquid chromatography–mass spectrometry/mass spectrometry
LITPOMS: ligand‐titration, fast photochemical oxidation of proteins and mass spectrometry
mAb: monoclonal antibody
MALDI: matrix‐assisted laser desorption ionization
Mas: mastoparan
Mel: melittin
MS: mass spectrometry
NMR: nuclear magnetic resonance
PLIMSTEX: protein–ligand interactions in solution by MS, titration, and, H/D exchange
SASA: solvent accessible surface area
SIMSTEX: self‐association interactions using mass spectrometry, self‐titration and H/D exchange
SK‐MLCK: skeletal muscle myosin light chain kinase
VKOR: vitamin K epoxide reductase
WT: wild type
